# Synthesis, crystal structure and characterizations of di-μ-cyanido-1:2κ^2^ *N*:*C*;2:3κ^2^ *C*:*N*-bis­(4,7,13,16,21,24-hexa­oxa-1,10-di­aza­bicyclo­[8.8.8]hexacosa­ne)-1κ^8^ *N* ^1^,*N* ^10^,*O* ^4^,*O* ^7^,*O* ^13^,*O* ^16^,*O* ^21^,*O* ^24^;3κ^8^ *N* ^1^,*N* ^10^,*O* ^4^,*O* ^7^,*O* ^13^,*O* ^16^,*O* ^21^,*O* ^24^-[5,10,15,20-tetra­kis­(4-chloro­phen­yl)porphyrinato-2κ^4^ *N*]-2-iron(II)-1,3-dipotassium(I) tetra­hydro­furan disolvate with an unknown solvent

**DOI:** 10.1107/S2056989019014841

**Published:** 2019-11-26

**Authors:** Tingting Huang, Haimang Wang, Jianping Zhao

**Affiliations:** a College of Material Science and Opto-electronic Technology, University of Chinese Academy of Sciences, Yanqi Lake, Huairou District, Beijing 101408, People’s Republic of China

**Keywords:** crystal structure, cyanide, iron(II), porphyrin, UV, FTIR

## Abstract

The title compound, [Fe(C_44_H_24_N_8_Cl_4_)(CN)_2_][K_2_(C_18_H_36_N_2_O_6_)_2_]·2C_4_H_8_O was synthesized and characterized by single-crystal X-ray diffraction as well as FTIR and UV–vis spectroscopy. The central Fe^II^ ion is coordinated by four pyrrole N atoms of the porphyrin core and two C atoms of the cyano groups in a slightly distorted octa­hedral coordination environment. The complex mol­ecule crystallizes with two tetra­hydro­furan solvent mol­ecules, one of which was refined as disordered over two sets of sites with refined occupancies of 0.619 (5) and 0.381 (5). It has a distorted porphyrin core with mean absolute core-atom displacements C_a_, C_b_, C_m_ and C_av_ of 0.32 (3), 0.22 (3), 0.56 (2) and 0.37 (14) Å, respectively. The axial Fe—C_cyano_ bond lengths are 1.991 (2) and 1.988 (2) Å. The average Fe—N_p_ (N_p_ is a porphyrin N atom) bond length is 1.964 (10) Å. One of the O atoms and several C atoms of the 222 moiety [222 = 4,7,13,16,21,24-hexa­oxa-1,10-di­aza­bicyclo­[8.8.8]hexa­cosa­ne] were refined as disordered over two sets of sites with occupancy ratios of 0.739 (6):0.261 (6) and 0.832 (4):0.168 (4). Additional solvent mol­ecules were found to be highly disordered and their contribution to the scattering was removed using the SQUEEZE procedure in *PLATON* [Spek (2015 ▸). *Acta Cryst*. C**71**, 9–18], which indicated a solvent cavity of volume 372 Å^3^ containing approximately 83 electrons. These solvent mol­ecules are not considered in the given chemical formula and other crystal data.

## Abstract 2

In the title compound, the central Fe^II^ ion is coordinated by four pyrrole N atoms of the porphyrin core and two C atoms of the cyano groups in a slightly distorted octa­hedral coordination environment. The complex mol­ecule has a distorted porphyrin core.

## Chemical context   

The cyanide ion, CN^−^, a well-known acute chemical poison, acts by inhibiting the enzyme cytochrome *c* oxidase, which catalyses the conversion of O_2_ to H_2_O along with the captured biological energy necessary to sustain life (Li *et al.*, 2015 ▸). It is often used as a ligand in ferric heme proteins in order to prepare low-spin (*S* = 1/2) ferric derivatives. These studies have raised questions about the geometry of the CN^−^ ligand when bound to iron in proteins (Schappacher *et al.*, 1989 ▸). The first cyano iron porphyrin structure, bis­(*tert*-butyl­isocyan­ide)octa­ethyl­oxophlorinatoiron(II), was reported by Jameson & Ibers (1979 ▸). However, since the reaction of cyanide with ferrohemes has been relatively little studied owing to the low stability of the complexes even at alkaline pH values (up to 9.4) (Yoshikawa *et al.*, 1985 ▸), only seven low-spin bis­(cyano)­iron(II) porphyrinates have since been characterized. Herein, the crystal structure of an iron(II) porphyrin complex, [K(222)]_2_[Fe^II^(T*p*ClPP)(CN)_2_]·2THF is reported where T*p*ClPP is 5,10,15,20-tetra­kis­(*p*-chloro­phen­yl) porphyrinato-*κ* ^4^ *N* and 222 is 4,7,13,16,21,24-hexa­oxa-1,10-di­aza­bicyclo[8.8.8]hexa­cosane and is used to stabilize the K^+^ cation.
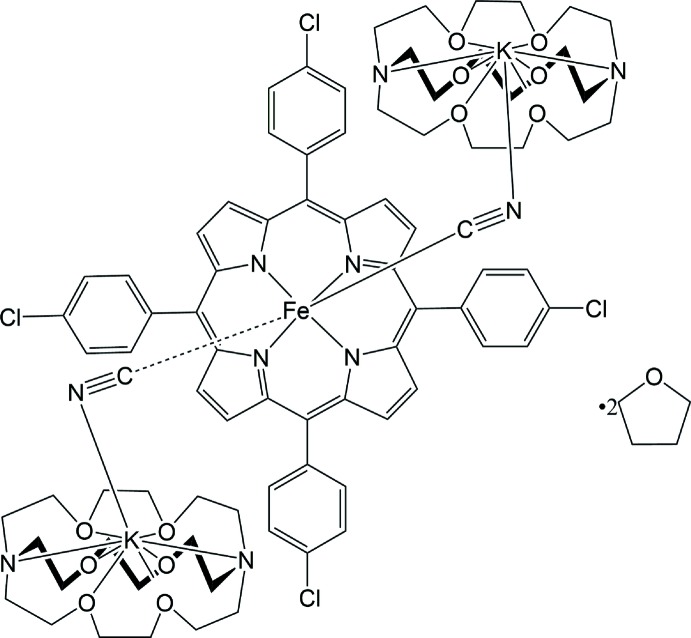


## Structural commentary   

**Figure 1:**
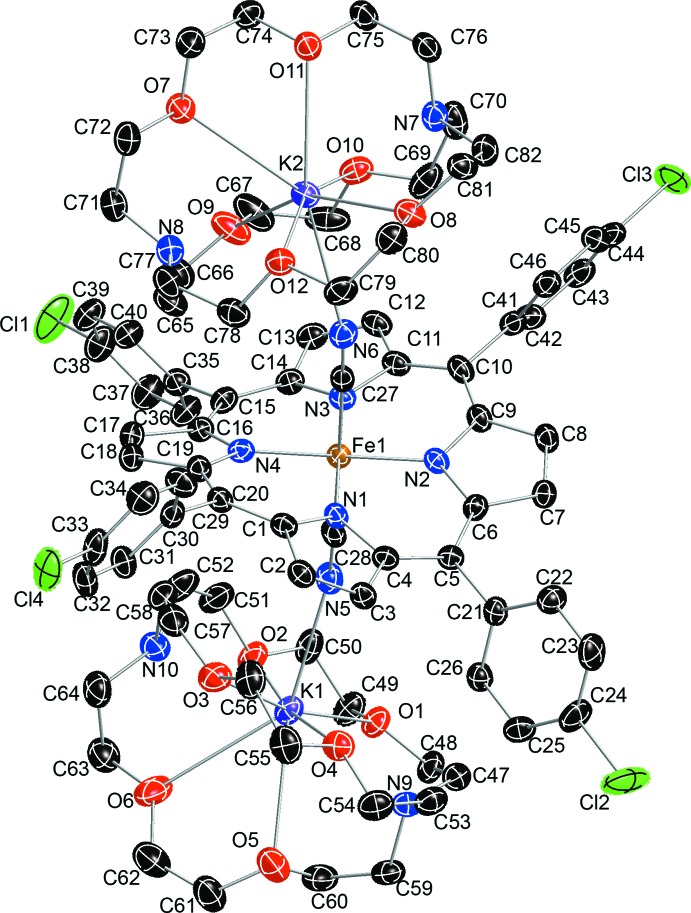
The mol­ecular structure of the title compound, with displacement ellipsoids drawn at the 50% probability level. For the sake of clarity, hydrogen atoms and solvent mol­ecule have been omitted and only the major components of disorder are shown.

**Figure 2:**
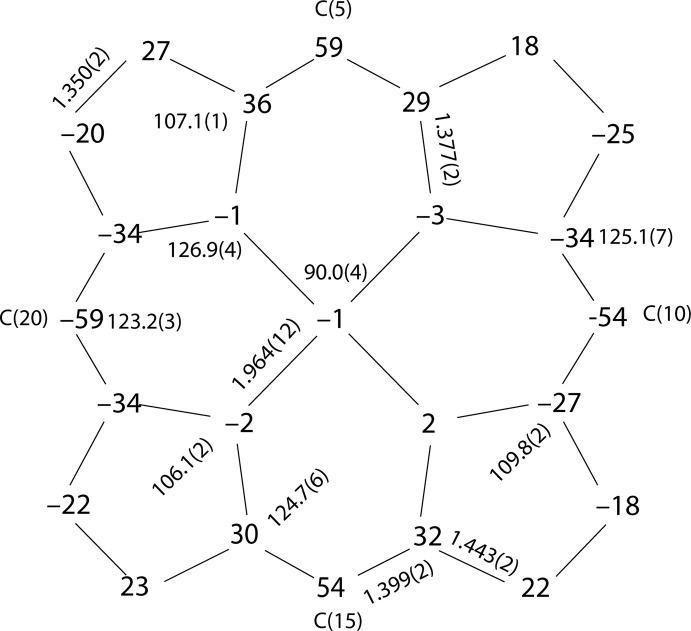
Scheme of the porphyrin core of the title compound. Averaged values of the chemically unique bond lengths (in Å) and angles (in °) are shown. The numbers in parentheses are the s.u. values calculated on the assumption that the averaged values are all drawn from the same population. The perpendicular displacements (in units of 0.01 Å) of the porphyrin core atoms from the 24-atom mean plane are also displayed. Positive values of the displacement are towards the C atoms of the axial ligand.

**Figure 3:**
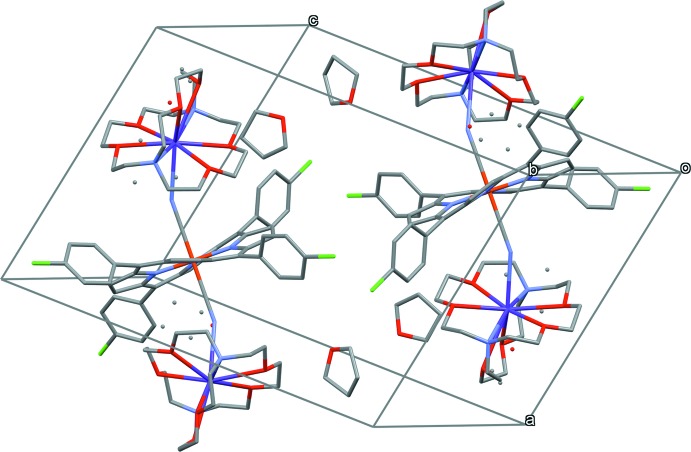
A view of the mol­ecular packing of the title compound in the crystal structure. Hydrogen atoms have been omitted for clarity. Unjoined atoms are the sites of the minor components of disorder.

In the title compound (Fig. 1 ▸), the asymmetric unit contains one six-coordinated iron(II) porphyrin in which the carbon atoms C27 and C28 of the cyanide ligands ligate to the central Fe^II^ ion, two cyano-bound [K(222)]^+^ ligands and two tetra­hydro­furan solvent mol­ecules. Additional qu­anti­tative information on the structure is given in Fig. 2 ▸, which shows the displacement of each porphyrin core atom (in units of 0.01 Å) from the 24-atom mean plane. Averaged values of the chemically unique bond lengths (in Å) and angles (in °) are also shown. The average Fe—N_p_ (N_p_ is the porphyrin nitro­gen atom) bond length is 1.964 (10) Å, similar to the distances in other reported bis­(cyano)­iron(II) porphyrinates [1.967 (12)–2.004 (5) Å; Li *et al.*, 2007 ▸). The mean axial Fe—C(cyano) bond length is 1.990 (2) Å, similar to 1.990 (5) Å for [PPN][Fe(TMP)(CN)_2_] (Bartczak *et al.*, 1998 ▸). The mean ligand C≡N bond length is 1.160 (1) Å. The average Fe—C—N angle involving the cyanide C and N atoms is nearly linear at 178.7 (1)°. The Fe^II^ ion is displaced slightly from the porphyrin core towards the axial ligand, as illustrated by the displacement of the metal atom from the 24-atom mean plane. The title compound shows a distorted porphyrin core conformation. The mean absolute core-atom displacements C_a_, C_b_, C_m_ and C_av_ are 0.32 (3), 0.22 (3), 0.56 (2) and 0.37 (14) Å, respectively. The mol­ecular packing is shown in Fig. 3 ▸.

## Supra­molecular features   

**Figure 4:**
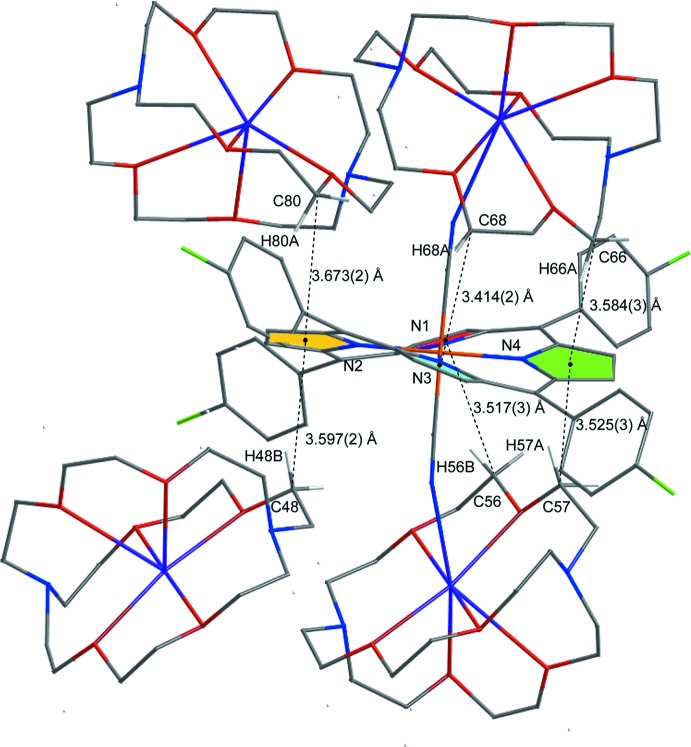
Diagram showing the distances between carbon atoms of [K(222)]^+^ and the centroids of pyrrole rings, which are involved in weak C—H⋯π inter­actions.

In order to represent clearly the inter­actions between [K(222)]^+^ and the porphyrin core in the title compound, the distances between the carbon atoms (hydrogen atoms) of [K(222)]^+^ and the close pyrrole ring centroids are shown in Fig. 4 ▸, and the geometrical parameters are listed in Table 1 ▸; all are in the range of C—H⋯π inter­actions (Takahashi *et al.*, 2001 ▸).

## Database survey   

In order to to compare the previously published structures of bis­(cyano)­iron porphyrinates that are potential biological models of heme protein a search of the Cambridge Structural Database (CSD version 5.39, update of August 2018; Groom *et al.*, 2016 ▸\bbr02s) was undertaken. This gave 26 hits for both iron(II) and iron(III) porphyrinates, but only eight structures [CSD refcodes: MUNGUZ (Nasri *et al.*, 2016 ▸), FURPAK, FURPOY and FURPEO (Li *et al.*, 2009 ▸), GUJSUA (Patra & Rath, 2009 ▸), FAJKUX (Rath *et al.*, 2004 ▸), POZDEN (Bartczak *et al.*, 1998 ▸), BITPFE (Jameson & Ibers, 1979 ▸)] are iron(II) porphyrinates. Selected bond lengths and bond angles in the title compound and related compounds with bis­(cyano) ligands are shown in Table 2 ▸.

## Characterization   

### FTIR spectroscopy   

**Figure 5:**
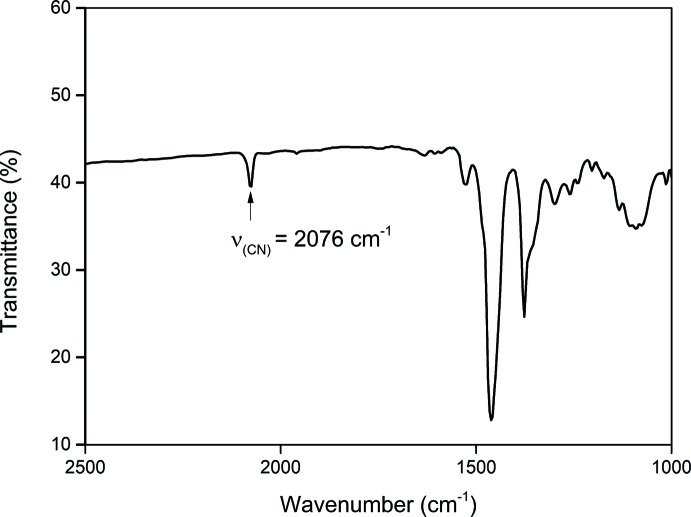
IR spectrum of the title compound.

The FTIR spectra were recorded on a Nicolet 6700 spectrometer as Nujol mulls. The IR spectroscopy of the title compound (KBr, cm^−1^) is shown in Fig. 5 ▸. Strong C—N bond-stretching frequencies of the cyanide ligand were observed at 2076 cm^−1^, which is comparable with reported values (He *et al.*, 2016 ▸; Scheidt *et al.*, 1983 ▸.)

### UV–vis titration   

**Figure 6:**
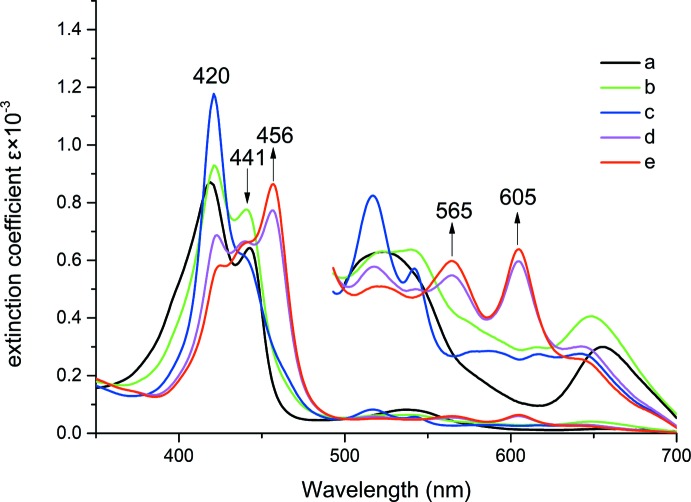
Selected UV–vis spectra taken in tetra­hydro­furan under argon. (*a*) [Fe^II^(T*p*ClPP)] (6.0 × 10 ^−5^ mol L^−1^); (*b*) [Fe^II^(T*p*ClPP)] in 2.4 × 10 ^−5^ mol L^−1^ [K(222)(CN)] solution; (*c*) [Fe^II^(T*p*ClPP)] in 6.0 × 10 ^−5^ mol L^−1^ [K(222)(CN)] solution; (*d*) [Fe^II^(T*p*ClPP)] in 8.4×10 ^−5^ mol L^−1^ [K(222)(CN)] solution; (*e*) [Fe^II^(T*p*ClPP)] in 1.2 × 10 ^−4^mol L^−1^ [K(222)(CN)] solution. The enlarged spectra from 500 to 750 nm were measured in a 10 mm UV cell.

UV–vis spectra were recorded on a PerkinElmer Lambda 19 UV/vis/near-IR spectrometer in a specially designed combined 1 and 10 mm inert-atmosphere cell. A solution of the four-coordinate iron(II) porphyrin was prepared using THF as solvent. The [K(222)(CN)] ligand solution was prepared by dissolving equivalent amounts of KCN and Kryptofix 222 in THF, the concentrations for UV–vis measurements being 0.02 mol L^−1^. The [K(222)(CN)] solution was titrated into an [Fe^II^(T*p*ClPP)] solution and the UV–vis spectra of [Fe^II^(T*p*ClPP)] were measured in different concentrations of [K(222)(CN)]. As shown in Fig. 6 ▸, the reaction suggests the presence of two cyano species, [Fe(T*p*ClPP)CN]^−^ and [Fe(T*p*ClPP)(CN)_2_]^2–^; both five-coordinated [Fe(T*p*ClPP)CN]^−^ and six-coordinated [Fe(T*p*ClPP)(CN)_2_]^2–^ can be isolated (Li *et al.*, 2009 ▸). Here, we have isolated six-coordinate [K(222)]_2_[Fe^II^(T*p*ClPP)(CN)_2_].

## Synthesis and crystallization   

### General procedure   

All reactions were performed using standard Schlenk techniques unless otherwise specified. All solvents were freeze/pump/thaw/degassed prior to use. Tetra­hydro­furan was refluxed in the presence of sodium and benzo­phenone under argon until the solution was blue. Hexanes (Beijing Chemical Works) were stored over potassium-sodium alloy and chloro­benzene (Sinopharm Chemical Reagent) over P_2_O_5_ (Sinopharm Chemical Reagent) under nitro­gen. 2,6-di­methyl­pyridine (Aladdin) and ethane­thiol (Aladdin) were purified by distillation before use. KCN was recrystallized and purified by a literature procedure (Armarego *et al.*, 2009 ▸). Krypotofix 222 (ACROS) was purified by vacuum sublimation. H_2_(T*p*ClPP), [Fe^III^(T*p*ClPP)Cl] and [Fe^III^(T*p*ClPP)]_2_O were prepared according to literature methods (Adler *et al.*, 1967 ▸; Fleischer & Srivastava, 1969 ▸) .

### Synthesis of the title compound   

The purple powder [Fe^III^(T*p*ClPP)]_2_O (10.0 mg, 0.006 mmol) was dried under vacuum for 1 h in a Schlenk tube. Chloro­benzene (∼5 mL) was transferred into the Schlenk tube by cannula and ethane thiol (∼2 mL, 0.028 mol) was added *via* syringe. The mixture was stirred under argon at ambient temperature for 48 h. Vacuum evaporation of the solvent yielded a dark-purple solid to which [K(222)(CN)] (0.012 mmol) in THF (∼8 mL) was added by cannula and the mixture was stirred overnight. X-ray quality crystals were obtained in 8 mm × 500 mm sealed glass tubes by liquid diffusion using hexa­nes as non-solvent.

## Refinement   

Crystal data, data collection and structure refinement details are summarized in Table 3 ▸. All hydrogen atoms were placed in calculated positions (C—H = 0.95, 0.98 and 0.99 Å for aryl, methyl and methlyene H atoms, respectively) and refined using a riding model with *U*_iso_(H) = 1.5*U*_eq_(C) for methyl H atoms or *U*_iso_(H) = 1.2*U*_eq_(C) otherwise. One of the THF mol­ecules is disordered and was modelled over two sets of sites with relative occupancies of 0.619 (5) and 0.381 (5). One of the O atoms and several C atoms of one of the 222 mol­ecules were refined as disordered over two sets of sites with refined occupancy ratios of 0.739 (6):0.261 (6) for O6/O6*A*, C61/C61*A*, C62/C62*A* and 0.832 (4):0.168 (4) for C52/C52*A*, C58/C58*A*, C64/C64*A*. Five reflections that were obscured by the beam stop were omitted in the last cycles of refinement. A region of electron density, most probably disordered THF possibly overlain with hexane, occupying voids of *ca* 372 Å^3^ for an electron count of 83, was removed with the SQUEEZE procedure in *PLATON* (Spek, 2015 ▸) following unsuccessful attempts to model it as plausible solvent mol­ecules. The stated formula mass, density, *etc*. do not include the disordered solvent

## Supplementary Material

Crystal structure: contains datablock(s) I, global. DOI: 10.1107/S2056989019014841/lh5930sup1.cif

Structure factors: contains datablock(s) I. DOI: 10.1107/S2056989019014841/lh5930Isup2.hkl

CCDC reference: 1967008

Additional supporting information:  crystallographic information; 3D view; checkCIF report

## Figures and Tables

**Table 1:** C—H⋯π inter­action geometry (Å, °). *Cg*1, *Cg*2, *Cg*3 and *Cg*4 are the centroids of atoms N1/C1–C4, N2/C6–C9, N3/C11–C14 and N4/C16–C19, respectively.

*D*—H⋯*A*	*D*—H	H⋯*A*	*D*⋯*A*	*D*—H⋯*A*
C48–H48*B*⋯*Cg*2^i^	0.99	2.75	3.597 (2)	143
C56–H56*B*⋯*Cg*1	0.99	2.69	3.517 (3)	141
C57–H57*A*⋯*Cg*4	0.99	2.75	3.525 (3)	135
C66–H66*A*⋯*Cg*4	0.99	2.79	3.584 (3)	138
C68–H68*A*⋯*Cg*3	0.99	2.90	3.414 (2)	114
C80–H80*A*⋯*Cg*2^ii^	0.99	2.90	3.673 (2)	135
C57*A*–H57*C*⋯*Cg*4	0.99	2.78	3.525 (3)	132

Symmetry codes: (i) 1 − *x*, 1 − *y*, −*z*; (ii) −*x*, 1 − *y*, 1 − *z*.

**Table 2:** Comparison of selected bond lengths and angles (Å, °) in the title compound with those in related compounds with bis­(cyano) ligands

Compound	Fe—C	Fe—Np	C—N	Fe—C—N
(1)	1.990 (2)	1.964 (10)	1.160 (1)	178.7 (1)
FAJKUX*^*a*^*	1.895	2.013 (5)	1.156	168.13
FURPAK*^*b*^*	2.068	2.067 (8)	1.100	176.51
FURPOY*^*b*^*	1.977	1.999 (5)	1.159	176.86
FURPEO*^*b*^*	1.969 (15)	1.991 (3)	1.157 (5)	176.86 (1)
GUJSUA*^*c*^*	1.989 (12)	1.982 (3)	1.141 (1)	179.65 (1)
MUNGUZ*^*c*^*	1.907	2.007 (1)	1.153	170.55

Notes: (*a*) Rath *et al.* (2004 ▸); (*b*) Li *et al.* (2009 ▸); (*c*) Patra & Rath (2009 ▸); (*d*) Nasri *et al.* (2016 ▸).

**Table 3:** Experimental details

Crystal data	
Chemical formula	[FeK_2_(C_44_H_24_Cl_4_N_4_)(C_18_H_36_N_2_O_6_)_2_(CN)_2_]·2C_4_H_8_O
*M*_r_	1833.74
Crystal system, space group	Triclinic, *P* 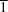
Temperature (K)	100
*a*, *b*, *c* (Å)	14.4457 (5), 17.6374 (7), 19.7411 (7)
α, β, γ (°)	76.267 (1), 79.587 (1), 80.311 (1)
*V* (Å^3^)	4763.9 (3)
*Z*	2
Radiation type	Mo *K*α
μ (mm^−1^)	0.42
Crystal size (mm)	0.63 × 0.35 × 0.25

Data collection	
Diffractometer	Bruker CCD area detector
Absorption correction	Multi-scan (*SADABS*; Bruker, 2014 ▸)
*T*_min_, *T*_max_	0.854, 0.896
No. of measured, independent and observed [*I* > 2σ(*I*)] reflections	103887, 20227, 16162
*R*_int_	0.047
(sin θ/λ)_max_ (Å^−1^)	0.634

Refinement	
*R*[*F* ^2^ > 2σ(*F* ^2^)], *wR*(*F* ^2^), *S*	0.044, 0.115, 1.02
No. of reflections	20227
No. of parameters	1193
No. of restraints	206
H-atom treatment	H-atom parameters constrained
Δρ_max_, Δρ_min_ (e Å^−3^)	0.98, −0.76

Computer programs: *APEX2* and *SAINT* (Bruker, 2014 ▸), *SHELXT2018/3* (Sheldrick, 2015*a* ▸), *SHELXL2018/3* (Sheldrick, 2015*b* ▸) and *OLEX2* (Dolomanov *et al.*, 2009 ▸).

## supplementary crystallographic information

### Crystal data

**Table:**

[FeK_2_(C_44_H_24_Cl_4_N_4_)(C_18_H_36_N_2_O_6_)_2_(CN)_2_]·2C_4_H_8_O	*Z* = 2
*M**_r_* = 1833.74	*F*(000) = 1932
Triclinic, *P*1	*D*_x_ = 1.278 Mg m^−^^3^
*a* = 14.4457 (5) Å	Mo *K*α radiation, λ = 0.71073 Å
*b* = 17.6374 (7) Å	Cell parameters from 1453 reflections
*c* = 19.7411 (7) Å	θ = 2.3–23.6°
α = 76.267 (1)°	µ = 0.42 mm^−^^1^
β = 79.587 (1)°	*T* = 100 K
γ = 80.311 (1)°	Prism, purple
*V* = 4763.9 (3) Å^3^	0.63 × 0.35 × 0.25 mm

### Data collection

**Table:**

Bruker CCD area detector diffractometer	16162 reflections with *I* > 2σ(*I*)
f\ and ω scans	*R*_int_ = 0.047
Absorption correction: multi-scan (SADABS; Bruker, 2014)	θ_max_ = 26.8°, θ_min_ = 1.5°
*T*_min_ = 0.854, *T*_max_ = 0.896	*h* = −17→18
103887 measured reflections	*k* = −22→22
20227 independent reflections	*l* = −24→24

### Refinement

**Table:**

Refinement on *F*^2^	Hydrogen site location: inferred from neighbouring sites
Least-squares matrix: full	H-atom parameters constrained
*R*[*F*^2^ > 2σ(*F*^2^)] = 0.044	*w* = 1/[σ^2^(*F*_o_^2^) + (0.0438*P*)^2^ + 5.8106*P*] where *P* = (*F*_o_^2^ + 2*F*_c_^2^)/3
*wR*(*F*^2^) = 0.115	(Δ/σ)_max_ = 0.001
*S* = 1.02	Δρ_max_ = 0.98 e Å^−^^3^
20227 reflections	Δρ_min_ = −0.76 e Å^−^^3^
1193 parameters	Extinction correction: SHELXL2018/3 (Sheldrick 2015b), Fc^*^=kFc[1+0.001xFc^2^λ^3^/sin(2θ)]^-1/4^
206 restraints	Extinction coefficient: 0.0023 (3)

### Special details

**Table:**

Geometry. All esds (except the esd in the dihedral angle between two l.s. planes) are estimated using the full covariance matrix. The cell esds are taken into account individually in the estimation of esds in distances, angles and torsion angles; correlations between esds in cell parameters are only used when they are defined by crystal symmetry. An approximate (isotropic) treatment of cell esds is used for estimating esds involving l.s. planes.

### Fractional atomic coordinates and isotropic or equivalent isotropic displacement parameters (Å^2^)

**Table:**

	*x*	*y*	*z*	*U*_iso_*/*U*_eq_	Occ. (<1)
Fe1	0.22990 (2)	0.35791 (2)	0.27285 (2)	0.01402 (7)	
K1	0.59481 (3)	0.30233 (3)	0.09438 (2)	0.02195 (10)	
K2	−0.12243 (3)	0.31486 (3)	0.48416 (2)	0.02041 (10)	
Cl1	0.09641 (6)	−0.01952 (4)	0.05513 (4)	0.0523 (2)	
Cl2	0.61872 (5)	0.73224 (5)	0.27178 (5)	0.0612 (2)	
Cl3	−0.25640 (4)	0.71962 (4)	0.07862 (4)	0.04068 (16)	
Cl4	0.48344 (5)	0.05205 (4)	0.67034 (3)	0.04017 (16)	
O1	0.58317 (10)	0.39629 (9)	−0.03794 (7)	0.0227 (3)	
O2	0.52382 (11)	0.24488 (9)	−0.00507 (8)	0.0268 (3)	
O3	0.57270 (11)	0.22260 (10)	0.22938 (8)	0.0290 (3)	
O4	0.63734 (11)	0.37469 (9)	0.19348 (8)	0.0269 (3)	
O5	0.81692 (14)	0.29449 (12)	0.07063 (13)	0.0607 (6)	
O6	0.7443 (3)	0.1467 (2)	0.1113 (2)	0.0472 (10)	0.739 (6)
O6A	0.7457 (8)	0.1538 (6)	0.0790 (7)	0.076 (3)	0.261 (6)
O7	−0.21004 (11)	0.16246 (9)	0.54843 (8)	0.0299 (4)	
O8	−0.11967 (10)	0.44807 (9)	0.53711 (8)	0.0239 (3)	
O9	−0.05999 (12)	0.21898 (11)	0.39318 (9)	0.0354 (4)	
O10	−0.17608 (11)	0.36143 (9)	0.34792 (8)	0.0256 (3)	
O11	−0.33128 (10)	0.30928 (9)	0.53000 (8)	0.0257 (3)	
O12	−0.01503 (10)	0.30007 (9)	0.59327 (8)	0.0255 (3)	
N1	0.31838 (11)	0.36548 (9)	0.33403 (8)	0.0149 (3)	
N2	0.21076 (11)	0.47357 (9)	0.24339 (8)	0.0149 (3)	
N3	0.14314 (11)	0.35119 (10)	0.20939 (8)	0.0169 (3)	
N4	0.24863 (11)	0.24203 (10)	0.30364 (8)	0.0159 (3)	
N5	0.39984 (13)	0.34516 (11)	0.14963 (10)	0.0263 (4)	
N6	0.06152 (13)	0.36455 (10)	0.39825 (9)	0.0231 (4)	
N7	−0.26794 (13)	0.46139 (11)	0.44956 (10)	0.0238 (4)	
N8	−0.00667 (13)	0.15980 (11)	0.52740 (10)	0.0282 (4)	
N9	0.69593 (12)	0.44838 (11)	0.04327 (9)	0.0220 (4)	
N10	0.53992 (13)	0.13835 (11)	0.13087 (10)	0.0286 (4)	
C1	0.34801 (13)	0.30831 (11)	0.38921 (10)	0.0166 (4)	
C2	0.41401 (14)	0.33656 (12)	0.42089 (10)	0.0190 (4)	
H2	0.442801	0.309162	0.460977	0.023*	
C3	0.42724 (14)	0.40943 (12)	0.38283 (10)	0.0180 (4)	
H3	0.469170	0.442048	0.389846	0.022*	
C4	0.36561 (13)	0.42839 (11)	0.32942 (10)	0.0160 (4)	
C5	0.34624 (13)	0.50323 (11)	0.28721 (10)	0.0158 (4)	
C6	0.26733 (14)	0.52549 (11)	0.25148 (10)	0.0159 (4)	
C7	0.22961 (14)	0.60489 (12)	0.22158 (10)	0.0181 (4)	
H7	0.256513	0.651285	0.218910	0.022*	
C8	0.14871 (14)	0.60094 (12)	0.19803 (10)	0.0190 (4)	
H8	0.106744	0.644223	0.177368	0.023*	
C9	0.13793 (14)	0.51877 (12)	0.21011 (10)	0.0171 (4)	
C10	0.07187 (14)	0.48935 (12)	0.18260 (10)	0.0174 (4)	
C11	0.07966 (14)	0.41065 (12)	0.17823 (10)	0.0177 (4)	
C12	0.02623 (14)	0.37955 (12)	0.13835 (10)	0.0208 (4)	
H12	−0.023883	0.407833	0.113587	0.025*	
C13	0.06067 (15)	0.30297 (13)	0.14280 (10)	0.0214 (4)	
H13	0.041127	0.267650	0.120230	0.026*	
C14	0.13287 (14)	0.28455 (12)	0.18828 (10)	0.0187 (4)	
C15	0.17678 (14)	0.20836 (12)	0.21321 (10)	0.0183 (4)	
C16	0.22683 (14)	0.18816 (12)	0.27072 (10)	0.0180 (4)	
C17	0.25524 (14)	0.10898 (12)	0.30820 (11)	0.0205 (4)	
H17	0.251038	0.061507	0.294813	0.025*	
C18	0.28888 (14)	0.11541 (12)	0.36589 (11)	0.0200 (4)	
H18	0.309664	0.073274	0.402055	0.024*	
C19	0.28732 (14)	0.19829 (11)	0.36224 (10)	0.0171 (4)	
C20	0.32848 (14)	0.23045 (11)	0.40592 (10)	0.0168 (4)	
C21	0.41118 (14)	0.56170 (11)	0.28457 (10)	0.0170 (4)	
C22	0.38165 (16)	0.63104 (13)	0.30878 (11)	0.0241 (4)	
H22	0.317237	0.642725	0.328494	0.029*	
C23	0.44570 (18)	0.68353 (14)	0.30438 (13)	0.0329 (5)	
H23	0.424937	0.730952	0.320730	0.040*	
C24	0.53888 (17)	0.66644 (14)	0.27632 (13)	0.0307 (5)	
C25	0.57112 (15)	0.59788 (13)	0.25235 (11)	0.0236 (4)	
H25	0.635854	0.586656	0.233146	0.028*	
C26	0.50721 (14)	0.54564 (12)	0.25683 (10)	0.0198 (4)	
H26	0.528866	0.498103	0.240821	0.024*	
C27	0.33719 (14)	0.35090 (11)	0.19480 (10)	0.0176 (4)	
C28	0.12304 (14)	0.36286 (11)	0.35168 (10)	0.0163 (4)	
C29	0.36380 (14)	0.17998 (11)	0.47106 (10)	0.0185 (4)	
C30	0.32023 (16)	0.19394 (13)	0.53663 (11)	0.0265 (5)	
H30	0.265153	0.231714	0.539174	0.032*	
C31	0.44429 (16)	0.12474 (12)	0.46852 (11)	0.0233 (4)	
H31	0.474518	0.113617	0.424407	0.028*	
C32	0.48137 (17)	0.08541 (13)	0.52963 (12)	0.0274 (5)	
H32	0.537196	0.048390	0.527266	0.033*	
C33	0.43626 (17)	0.10070 (13)	0.59368 (11)	0.0265 (5)	
C34	0.35496 (18)	0.15421 (14)	0.59848 (12)	0.0306 (5)	
H34	0.323602	0.163610	0.642924	0.037*	
C35	0.16158 (14)	0.14654 (12)	0.17767 (11)	0.0202 (4)	
C36	0.19681 (16)	0.15292 (13)	0.10635 (11)	0.0260 (5)	
H36	0.234601	0.193335	0.083007	0.031*	
C37	0.17802 (18)	0.10173 (14)	0.06872 (12)	0.0316 (5)	
H37	0.202607	0.106712	0.020098	0.038*	
C38	0.12294 (18)	0.04333 (14)	0.10309 (12)	0.0306 (5)	
C39	0.08771 (17)	0.03427 (14)	0.17399 (12)	0.0293 (5)	
H39	0.050622	−0.006720	0.196992	0.035*	
C40	0.10731 (15)	0.08605 (13)	0.21125 (11)	0.0238 (4)	
H40	0.083515	0.080179	0.260071	0.029*	
C41	−0.00842 (14)	0.54645 (12)	0.15494 (10)	0.0185 (4)	
C42	−0.02505 (14)	0.56031 (13)	0.08483 (11)	0.0222 (4)	
H42	0.016380	0.532659	0.052994	0.027*	
C43	−0.10061 (15)	0.61348 (13)	0.06109 (12)	0.0260 (5)	
H43	−0.110924	0.622498	0.013475	0.031*	
C44	−0.16054 (15)	0.65304 (13)	0.10786 (12)	0.0260 (5)	
C45	−0.14706 (15)	0.64136 (13)	0.17745 (12)	0.0253 (5)	
H45	−0.189211	0.668916	0.209029	0.030*	
C46	−0.07022 (14)	0.58827 (12)	0.20006 (11)	0.0210 (4)	
H46	−0.059713	0.580381	0.247515	0.025*	
C47	0.64176 (15)	0.50126 (13)	−0.00974 (12)	0.0252 (5)	
H47A	0.673846	0.548710	−0.030072	0.030*	
H47B	0.577719	0.518439	0.013757	0.030*	
C48	0.63176 (16)	0.46376 (13)	−0.06823 (11)	0.0250 (5)	
H48A	0.595159	0.501668	−0.102522	0.030*	
H48B	0.695168	0.447577	−0.093263	0.030*	
C49	0.56857 (18)	0.35789 (14)	−0.08965 (11)	0.0290 (5)	
H49A	0.627994	0.325204	−0.104708	0.035*	
H49B	0.550204	0.397379	−0.131430	0.035*	
C50	0.49153 (16)	0.30699 (14)	−0.05918 (12)	0.0276 (5)	
H50A	0.434085	0.338681	−0.039668	0.033*	
H50B	0.474854	0.285106	−0.096496	0.033*	
C51	0.45031 (18)	0.19920 (15)	0.03090 (14)	0.0360 (6)	0.832 (4)
H51A	0.417102	0.185737	−0.003495	0.043*	0.832 (4)
H51B	0.403437	0.229372	0.061084	0.043*	0.832 (4)
C52	0.4955 (2)	0.12458 (17)	0.07583 (17)	0.0375 (8)	0.832 (4)
H52A	0.446104	0.089871	0.097564	0.045*	0.832 (4)
H52B	0.543815	0.096428	0.044879	0.045*	0.832 (4)
C51A	0.45031 (18)	0.19920 (15)	0.03090 (14)	0.0360 (6)	0.168 (4)
H51C	0.452736	0.156583	0.005547	0.043*	0.168 (4)
H51D	0.389267	0.233417	0.024952	0.043*	0.168 (4)
C52A	0.4459 (6)	0.1620 (8)	0.1068 (4)	0.032 (3)	0.168 (4)
H52C	0.412872	0.114822	0.116646	0.038*	0.168 (4)
H52D	0.407470	0.199328	0.134586	0.038*	0.168 (4)
C53	0.68814 (17)	0.48300 (14)	0.10485 (12)	0.0278 (5)	
H53A	0.622619	0.509576	0.114600	0.033*	
H53B	0.731699	0.523431	0.093968	0.033*	
C54	0.71150 (17)	0.42287 (15)	0.17006 (12)	0.0309 (5)	
H54A	0.772668	0.390103	0.159078	0.037*	
H54B	0.717306	0.449761	0.207489	0.037*	
C55	0.65216 (19)	0.31670 (15)	0.25602 (12)	0.0347 (6)	
H55A	0.658182	0.342624	0.294046	0.042*	
H55B	0.711596	0.281081	0.246980	0.042*	
C56	0.56970 (19)	0.27031 (15)	0.27831 (12)	0.0340 (5)	
H56A	0.573121	0.236899	0.326014	0.041*	
H56B	0.509385	0.306510	0.279879	0.041*	
C57	0.50382 (18)	0.16933 (14)	0.25173 (13)	0.0342 (5)	0.832 (4)
H57A	0.449136	0.192885	0.281513	0.041*	0.832 (4)
H57B	0.531907	0.120279	0.281226	0.041*	0.832 (4)
C58	0.4689 (2)	0.14913 (17)	0.19284 (15)	0.0336 (7)	0.832 (4)
H58A	0.439784	0.099950	0.211398	0.040*	0.832 (4)
H58B	0.418083	0.191283	0.176865	0.040*	0.832 (4)
C57A	0.50382 (18)	0.16933 (14)	0.25173 (13)	0.0342 (5)	0.168 (4)
H57C	0.439556	0.198565	0.247732	0.041*	0.168 (4)
H57D	0.505797	0.142772	0.301712	0.041*	0.168 (4)
C58A	0.5243 (10)	0.1087 (5)	0.2064 (4)	0.030 (3)	0.168 (4)
H58C	0.581252	0.072250	0.219705	0.036*	0.168 (4)
H58D	0.470417	0.077592	0.217746	0.036*	0.168 (4)
C59	0.79571 (15)	0.42891 (14)	0.01273 (12)	0.0266 (5)	
H59A	0.837498	0.437677	0.043977	0.032*	
H59B	0.808763	0.465293	−0.033476	0.032*	
C60	0.82026 (18)	0.34591 (15)	0.00267 (15)	0.0389 (6)	
H60A	0.774376	0.333970	−0.023757	0.047*	
H60B	0.884610	0.338747	−0.024488	0.047*	
C61	0.8745 (3)	0.2209 (2)	0.0813 (2)	0.0389 (9)	0.739 (6)
H61A	0.881877	0.202893	0.131742	0.047*	0.739 (6)
H61B	0.938336	0.225914	0.053485	0.047*	0.739 (6)
C62	0.8289 (2)	0.1620 (2)	0.0587 (2)	0.0356 (10)	0.739 (6)
H62A	0.810793	0.183583	0.011121	0.043*	0.739 (6)
H62B	0.873260	0.112777	0.057501	0.043*	0.739 (6)
C61A	0.8543 (7)	0.2216 (4)	0.0384 (5)	0.040 (2)	0.261 (6)
H61C	0.818774	0.222087	−0.000134	0.048*	0.261 (6)
H61D	0.922346	0.221984	0.018773	0.048*	0.261 (6)
C62A	0.8413 (8)	0.1491 (7)	0.0956 (8)	0.053 (3)	0.261 (6)
H62C	0.884133	0.101531	0.086170	0.063*	0.261 (6)
H62D	0.842778	0.156724	0.143486	0.063*	0.261 (6)
C63	0.70504 (18)	0.08249 (15)	0.09836 (15)	0.0398 (6)	0.832 (4)
H63A	0.750387	0.033502	0.105225	0.048*	0.832 (4)
H63B	0.690133	0.094522	0.049614	0.048*	0.832 (4)
C64	0.6162 (2)	0.07372 (17)	0.15085 (15)	0.0334 (7)	0.832 (4)
H64A	0.594795	0.022575	0.153228	0.040*	0.832 (4)
H64B	0.629783	0.074119	0.198146	0.040*	0.832 (4)
C63A	0.70504 (18)	0.08249 (15)	0.09836 (15)	0.0398 (6)	0.168 (4)
H63C	0.710239	0.059889	0.148680	0.048*	0.168 (4)
H63D	0.744539	0.045279	0.070955	0.048*	0.168 (4)
C64A	0.6017 (5)	0.0851 (8)	0.0887 (7)	0.039 (3)	0.168 (4)
H64C	0.594825	0.104369	0.038316	0.046*	0.168 (4)
H64D	0.582789	0.031592	0.104306	0.046*	0.168 (4)
C65	0.06157 (17)	0.15839 (15)	0.46261 (13)	0.0339 (5)	
H65A	0.108430	0.110513	0.470918	0.041*	
H65B	0.096403	0.204528	0.452611	0.041*	
C66	0.01801 (17)	0.15935 (15)	0.39847 (13)	0.0338 (5)	
H66A	0.065711	0.169206	0.355632	0.041*	
H66B	−0.003081	0.107680	0.402485	0.041*	
C67	−0.09170 (19)	0.23932 (17)	0.32649 (14)	0.0400 (6)	
H67A	−0.153479	0.220228	0.330360	0.048*	
H67B	−0.045341	0.214281	0.292209	0.048*	
C68	−0.10201 (16)	0.32650 (17)	0.30179 (12)	0.0344 (6)	
H68A	−0.041537	0.346005	0.301532	0.041*	
H68B	−0.117546	0.341176	0.253142	0.041*	
C69	−0.18001 (18)	0.44470 (14)	0.33286 (12)	0.0312 (5)	
H69A	−0.179055	0.466035	0.281648	0.037*	
H69B	−0.124438	0.459451	0.347230	0.037*	
C70	−0.27039 (18)	0.47791 (14)	0.37312 (13)	0.0323 (5)	
H70A	−0.281360	0.535595	0.355273	0.039*	
H70B	−0.324265	0.455198	0.364518	0.039*	
C71	−0.05640 (17)	0.08982 (14)	0.54791 (14)	0.0335 (5)	
H71A	−0.016928	0.045996	0.575330	0.040*	
H71B	−0.064841	0.073693	0.504913	0.040*	
C72	−0.15153 (17)	0.10434 (14)	0.59115 (14)	0.0337 (5)	
H72A	−0.180723	0.054988	0.607151	0.040*	
H72B	−0.144516	0.122999	0.633227	0.040*	
C73	−0.30445 (17)	0.17179 (14)	0.58188 (13)	0.0321 (5)	
H73A	−0.306894	0.186779	0.627485	0.039*	
H73B	−0.331340	0.121625	0.591199	0.039*	
C74	−0.36121 (16)	0.23516 (13)	0.53442 (14)	0.0298 (5)	
H74A	−0.351793	0.223459	0.486830	0.036*	
H74B	−0.429618	0.236778	0.553484	0.036*	
C75	−0.39085 (16)	0.37104 (13)	0.49173 (13)	0.0278 (5)	
H75A	−0.457747	0.369378	0.514226	0.033*	
H75B	−0.385925	0.363865	0.442903	0.033*	
C76	−0.36163 (15)	0.44973 (13)	0.49039 (13)	0.0277 (5)	
H76A	−0.409374	0.492127	0.470043	0.033*	
H76B	−0.361252	0.454194	0.539389	0.033*	
C77	0.04196 (17)	0.16584 (14)	0.58476 (13)	0.0330 (5)	
H77A	0.100470	0.127461	0.585501	0.040*	
H77B	0.000330	0.151067	0.630167	0.040*	
C78	0.06825 (16)	0.24677 (14)	0.57889 (13)	0.0309 (5)	
H78A	0.112112	0.243983	0.612822	0.037*	
H78B	0.101151	0.265949	0.530800	0.037*	
C79	0.01044 (16)	0.37579 (13)	0.59082 (12)	0.0276 (5)	
H79A	0.049958	0.393985	0.545143	0.033*	
H79B	0.048216	0.371916	0.628931	0.033*	
C80	−0.07656 (17)	0.43359 (14)	0.59936 (12)	0.0276 (5)	
H80A	−0.121111	0.412085	0.641144	0.033*	
H80B	−0.059670	0.483195	0.605999	0.033*	
C81	−0.19935 (15)	0.50749 (13)	0.53796 (12)	0.0248 (4)	
H81A	−0.181635	0.555062	0.548115	0.030*	
H81B	−0.251242	0.488505	0.575063	0.030*	
C82	−0.23147 (17)	0.52675 (13)	0.46697 (13)	0.0278 (5)	
H82A	−0.281962	0.572421	0.465116	0.033*	
H82B	−0.177439	0.542612	0.430520	0.033*	
O13	0.13665 (19)	−0.03498 (16)	0.6175 (2)	0.0903 (10)	
C83	0.1504 (3)	−0.0366 (2)	0.6852 (2)	0.0736 (12)	
H83A	0.116221	−0.077337	0.719164	0.088*	
H83B	0.125901	0.015046	0.697466	0.088*	
C84	0.2549 (3)	−0.0552 (3)	0.6879 (2)	0.0790 (12)	
H84A	0.281015	−0.008810	0.693092	0.095*	
H84B	0.268443	−0.099608	0.727596	0.095*	
C85	0.2947 (3)	−0.0762 (3)	0.6203 (2)	0.0796 (12)	
H85A	0.356641	−0.056527	0.601941	0.096*	
H85B	0.303632	−0.134067	0.625023	0.096*	
C86	0.2256 (3)	−0.0387 (3)	0.5744 (2)	0.0952 (17)	
H86A	0.239677	0.014875	0.550002	0.114*	
H86B	0.226074	−0.069634	0.538550	0.114*	
O14	−0.1558 (3)	0.8094 (3)	0.25418 (19)	0.0721 (14)	0.619 (5)
C87	−0.0560 (5)	0.7966 (5)	0.2359 (4)	0.064 (2)	0.619 (5)
H87A	−0.031435	0.741556	0.256149	0.077*	0.619 (5)
H87B	−0.025977	0.832091	0.254840	0.077*	0.619 (5)
C88	−0.0327 (9)	0.8130 (9)	0.1563 (7)	0.059 (3)	0.619 (5)
H88A	−0.036184	0.766647	0.137118	0.071*	0.619 (5)
H88B	0.030355	0.831013	0.139380	0.071*	0.619 (5)
C89	−0.1135 (7)	0.8789 (6)	0.1390 (5)	0.071 (3)	0.619 (5)
H89A	−0.092956	0.930813	0.134850	0.085*	0.619 (5)
H89B	−0.133434	0.877833	0.093830	0.085*	0.619 (5)
C90	−0.1939 (5)	0.8651 (4)	0.1983 (3)	0.0677 (17)	0.619 (5)
H90A	−0.221084	0.914812	0.213447	0.081*	0.619 (5)
H90B	−0.244678	0.844359	0.183189	0.081*	0.619 (5)
O14A	−0.0533 (6)	0.8680 (4)	0.2346 (4)	0.078 (3)	0.381 (5)
C87A	−0.1265 (10)	0.8994 (9)	0.1936 (7)	0.095 (5)	0.381 (5)
H87C	−0.122363	0.955822	0.172868	0.114*	0.381 (5)
H87D	−0.188911	0.894884	0.223735	0.114*	0.381 (5)
C88A	−0.1171 (15)	0.8531 (7)	0.1334 (10)	0.074 (5)	0.381 (5)
H88C	−0.117490	0.888770	0.086414	0.089*	0.381 (5)
H88D	−0.167145	0.818480	0.141929	0.089*	0.381 (5)
C89A	−0.0160 (19)	0.8046 (15)	0.1426 (13)	0.076 (6)	0.381 (5)
H89C	−0.010678	0.752917	0.129840	0.091*	0.381 (5)
H89D	0.034962	0.833898	0.112970	0.091*	0.381 (5)
C90A	−0.0108 (11)	0.7950 (10)	0.2169 (8)	0.075 (4)	0.381 (5)
H90C	−0.045739	0.751637	0.245398	0.090*	0.381 (5)
H90D	0.056092	0.783160	0.225490	0.090*	0.381 (5)

### Atomic displacement parameters (Å^2^)

**Table:**

	*U*^11^	*U*^22^	*U*^33^	*U*^12^	*U*^13^	*U*^23^
Fe1	0.01386 (14)	0.01428 (14)	0.01392 (14)	−0.00143 (10)	−0.00411 (10)	−0.00175 (10)
K1	0.0226 (2)	0.0254 (2)	0.0163 (2)	−0.00555 (18)	−0.00272 (17)	0.00008 (17)
K2	0.0188 (2)	0.0229 (2)	0.0194 (2)	−0.00134 (17)	−0.00299 (16)	−0.00523 (17)
Cl1	0.0875 (6)	0.0472 (4)	0.0367 (3)	−0.0365 (4)	−0.0093 (3)	−0.0175 (3)
Cl2	0.0404 (4)	0.0610 (5)	0.0993 (6)	−0.0305 (3)	0.0126 (4)	−0.0511 (5)
Cl3	0.0251 (3)	0.0390 (3)	0.0505 (4)	0.0053 (2)	−0.0177 (3)	0.0075 (3)
Cl4	0.0579 (4)	0.0345 (3)	0.0282 (3)	−0.0111 (3)	−0.0248 (3)	0.0104 (2)
O1	0.0264 (8)	0.0232 (8)	0.0174 (7)	−0.0086 (6)	−0.0026 (6)	0.0011 (6)
O2	0.0265 (8)	0.0258 (8)	0.0273 (8)	−0.0097 (6)	−0.0095 (6)	0.0047 (6)
O3	0.0318 (9)	0.0316 (9)	0.0193 (7)	−0.0048 (7)	0.0036 (6)	−0.0020 (6)
O4	0.0280 (8)	0.0324 (9)	0.0193 (7)	−0.0042 (7)	−0.0055 (6)	−0.0020 (6)
O5	0.0357 (11)	0.0338 (11)	0.0903 (17)	0.0063 (8)	0.0049 (11)	0.0119 (11)
O6	0.0293 (13)	0.0442 (17)	0.066 (3)	−0.0106 (11)	0.0188 (17)	−0.0225 (18)
O6A	0.045 (4)	0.050 (4)	0.099 (6)	0.004 (3)	0.040 (5)	0.004 (5)
O7	0.0257 (8)	0.0263 (8)	0.0317 (9)	−0.0016 (6)	0.0021 (7)	−0.0005 (7)
O8	0.0221 (8)	0.0295 (8)	0.0229 (7)	−0.0002 (6)	−0.0053 (6)	−0.0116 (6)
O9	0.0317 (9)	0.0417 (10)	0.0365 (9)	0.0099 (7)	−0.0103 (7)	−0.0219 (8)
O10	0.0243 (8)	0.0324 (9)	0.0196 (7)	−0.0082 (6)	0.0009 (6)	−0.0043 (6)
O11	0.0211 (8)	0.0207 (8)	0.0384 (9)	−0.0021 (6)	−0.0112 (6)	−0.0081 (7)
O12	0.0211 (7)	0.0251 (8)	0.0311 (8)	−0.0042 (6)	−0.0061 (6)	−0.0049 (6)
N1	0.0155 (8)	0.0129 (8)	0.0156 (8)	−0.0006 (6)	−0.0038 (6)	−0.0013 (6)
N2	0.0143 (8)	0.0165 (8)	0.0133 (8)	−0.0004 (6)	−0.0036 (6)	−0.0019 (6)
N3	0.0163 (8)	0.0187 (8)	0.0156 (8)	−0.0027 (6)	−0.0032 (6)	−0.0025 (6)
N4	0.0139 (8)	0.0178 (8)	0.0159 (8)	−0.0022 (6)	−0.0036 (6)	−0.0024 (6)
N5	0.0267 (10)	0.0240 (10)	0.0233 (9)	0.0000 (7)	−0.0012 (8)	0.0002 (7)
N6	0.0236 (9)	0.0224 (9)	0.0239 (9)	−0.0046 (7)	−0.0018 (8)	−0.0065 (7)
N7	0.0231 (9)	0.0220 (9)	0.0292 (10)	−0.0038 (7)	−0.0094 (7)	−0.0062 (7)
N8	0.0269 (10)	0.0223 (9)	0.0343 (11)	−0.0017 (8)	−0.0063 (8)	−0.0036 (8)
N9	0.0173 (8)	0.0245 (9)	0.0240 (9)	−0.0037 (7)	−0.0021 (7)	−0.0052 (7)
N10	0.0264 (10)	0.0252 (10)	0.0292 (10)	−0.0017 (8)	−0.0026 (8)	0.0016 (8)
C1	0.0149 (9)	0.0184 (10)	0.0162 (9)	−0.0003 (7)	−0.0048 (7)	−0.0024 (7)
C2	0.0199 (10)	0.0200 (10)	0.0174 (9)	0.0002 (8)	−0.0069 (8)	−0.0035 (8)
C3	0.0177 (10)	0.0185 (10)	0.0198 (10)	−0.0024 (7)	−0.0064 (8)	−0.0052 (8)
C4	0.0141 (9)	0.0178 (9)	0.0167 (9)	−0.0016 (7)	−0.0039 (7)	−0.0037 (7)
C5	0.0156 (9)	0.0178 (9)	0.0137 (9)	−0.0020 (7)	−0.0013 (7)	−0.0038 (7)
C6	0.0176 (9)	0.0153 (9)	0.0141 (9)	−0.0024 (7)	−0.0013 (7)	−0.0024 (7)
C7	0.0206 (10)	0.0157 (9)	0.0167 (9)	−0.0022 (8)	−0.0034 (8)	−0.0008 (7)
C8	0.0186 (10)	0.0170 (10)	0.0188 (10)	0.0010 (8)	−0.0040 (8)	−0.0004 (8)
C9	0.0176 (9)	0.0191 (10)	0.0129 (9)	0.0001 (7)	−0.0038 (7)	−0.0007 (7)
C10	0.0157 (9)	0.0209 (10)	0.0133 (9)	0.0004 (8)	−0.0032 (7)	−0.0005 (7)
C11	0.0160 (9)	0.0223 (10)	0.0145 (9)	−0.0030 (8)	−0.0043 (7)	−0.0015 (8)
C12	0.0181 (10)	0.0263 (11)	0.0179 (10)	−0.0029 (8)	−0.0063 (8)	−0.0018 (8)
C13	0.0222 (10)	0.0272 (11)	0.0170 (10)	−0.0069 (8)	−0.0052 (8)	−0.0046 (8)
C14	0.0193 (10)	0.0223 (10)	0.0157 (9)	−0.0047 (8)	−0.0033 (8)	−0.0046 (8)
C15	0.0193 (10)	0.0186 (10)	0.0183 (9)	−0.0058 (8)	−0.0023 (8)	−0.0047 (8)
C16	0.0162 (9)	0.0176 (10)	0.0202 (10)	−0.0033 (7)	−0.0018 (8)	−0.0040 (8)
C17	0.0214 (10)	0.0153 (10)	0.0250 (10)	−0.0030 (8)	−0.0035 (8)	−0.0039 (8)
C18	0.0204 (10)	0.0162 (10)	0.0217 (10)	−0.0015 (8)	−0.0046 (8)	−0.0005 (8)
C19	0.0172 (9)	0.0150 (9)	0.0170 (9)	−0.0019 (7)	−0.0011 (7)	−0.0003 (7)
C20	0.0163 (9)	0.0160 (9)	0.0161 (9)	0.0001 (7)	−0.0026 (7)	−0.0013 (7)
C21	0.0212 (10)	0.0164 (9)	0.0137 (9)	−0.0030 (8)	−0.0067 (7)	−0.0005 (7)
C22	0.0241 (11)	0.0235 (11)	0.0259 (11)	−0.0032 (8)	−0.0020 (9)	−0.0088 (9)
C23	0.0362 (13)	0.0268 (12)	0.0411 (14)	−0.0085 (10)	−0.0010 (11)	−0.0180 (10)
C24	0.0292 (12)	0.0312 (12)	0.0379 (13)	−0.0146 (10)	−0.0037 (10)	−0.0133 (10)
C25	0.0202 (10)	0.0291 (11)	0.0231 (10)	−0.0062 (9)	−0.0040 (8)	−0.0062 (9)
C26	0.0222 (10)	0.0186 (10)	0.0190 (10)	−0.0024 (8)	−0.0057 (8)	−0.0029 (8)
C27	0.0187 (10)	0.0146 (9)	0.0193 (10)	−0.0011 (7)	−0.0078 (8)	−0.0004 (7)
C28	0.0173 (9)	0.0140 (9)	0.0193 (10)	−0.0020 (7)	−0.0075 (8)	−0.0034 (7)
C29	0.0199 (10)	0.0156 (9)	0.0201 (10)	−0.0052 (8)	−0.0062 (8)	0.0008 (8)
C30	0.0266 (11)	0.0282 (12)	0.0221 (11)	0.0012 (9)	−0.0047 (9)	−0.0027 (9)
C31	0.0279 (11)	0.0193 (10)	0.0218 (10)	0.0012 (8)	−0.0069 (8)	−0.0030 (8)
C32	0.0326 (12)	0.0175 (10)	0.0322 (12)	0.0013 (9)	−0.0133 (10)	−0.0021 (9)
C33	0.0381 (13)	0.0199 (11)	0.0225 (11)	−0.0089 (9)	−0.0143 (9)	0.0043 (8)
C34	0.0378 (13)	0.0351 (13)	0.0182 (10)	−0.0071 (10)	−0.0037 (9)	−0.0030 (9)
C35	0.0204 (10)	0.0201 (10)	0.0213 (10)	−0.0023 (8)	−0.0051 (8)	−0.0055 (8)
C36	0.0309 (12)	0.0259 (11)	0.0229 (11)	−0.0120 (9)	0.0000 (9)	−0.0059 (9)
C37	0.0424 (14)	0.0332 (13)	0.0224 (11)	−0.0119 (10)	−0.0016 (10)	−0.0095 (10)
C38	0.0420 (14)	0.0271 (12)	0.0299 (12)	−0.0110 (10)	−0.0097 (10)	−0.0122 (10)
C39	0.0329 (12)	0.0267 (12)	0.0314 (12)	−0.0136 (10)	−0.0050 (10)	−0.0050 (9)
C40	0.0247 (11)	0.0259 (11)	0.0219 (10)	−0.0075 (9)	−0.0019 (8)	−0.0053 (9)
C41	0.0152 (9)	0.0185 (10)	0.0205 (10)	−0.0047 (7)	−0.0048 (8)	0.0014 (8)
C42	0.0181 (10)	0.0266 (11)	0.0195 (10)	−0.0041 (8)	−0.0040 (8)	0.0012 (8)
C43	0.0214 (11)	0.0306 (12)	0.0242 (11)	−0.0083 (9)	−0.0102 (9)	0.0064 (9)
C44	0.0153 (10)	0.0242 (11)	0.0345 (12)	−0.0038 (8)	−0.0113 (9)	0.0079 (9)
C45	0.0180 (10)	0.0235 (11)	0.0306 (12)	−0.0010 (8)	−0.0016 (9)	−0.0008 (9)
C46	0.0202 (10)	0.0201 (10)	0.0205 (10)	−0.0026 (8)	−0.0058 (8)	0.0018 (8)
C47	0.0224 (11)	0.0228 (11)	0.0285 (11)	−0.0037 (8)	−0.0034 (9)	−0.0017 (9)
C48	0.0251 (11)	0.0235 (11)	0.0235 (11)	−0.0078 (9)	−0.0032 (9)	0.0036 (9)
C49	0.0380 (13)	0.0332 (13)	0.0169 (10)	−0.0137 (10)	−0.0072 (9)	0.0010 (9)
C50	0.0285 (12)	0.0299 (12)	0.0247 (11)	−0.0072 (9)	−0.0124 (9)	0.0025 (9)
C51	0.0317 (13)	0.0345 (13)	0.0402 (14)	−0.0158 (10)	−0.0103 (11)	0.0073 (11)
C52	0.0434 (18)	0.0275 (15)	0.0415 (17)	−0.0135 (13)	−0.0154 (14)	0.0061 (13)
C51A	0.0317 (13)	0.0345 (13)	0.0402 (14)	−0.0158 (10)	−0.0103 (11)	0.0073 (11)
C52A	0.037 (5)	0.035 (5)	0.029 (5)	−0.019 (5)	−0.011 (5)	−0.003 (5)
C53	0.0274 (12)	0.0290 (12)	0.0299 (12)	−0.0095 (9)	−0.0017 (9)	−0.0100 (9)
C54	0.0293 (12)	0.0398 (14)	0.0290 (12)	−0.0073 (10)	−0.0085 (9)	−0.0125 (10)
C55	0.0469 (15)	0.0355 (13)	0.0221 (11)	−0.0008 (11)	−0.0140 (10)	−0.0036 (10)
C56	0.0477 (15)	0.0326 (13)	0.0159 (10)	−0.0001 (11)	−0.0005 (10)	−0.0006 (9)
C57	0.0331 (13)	0.0291 (12)	0.0306 (12)	−0.0037 (10)	0.0087 (10)	0.0024 (10)
C58	0.0273 (15)	0.0258 (15)	0.0399 (16)	−0.0057 (12)	0.0026 (12)	0.0043 (12)
C57A	0.0331 (13)	0.0291 (12)	0.0306 (12)	−0.0037 (10)	0.0087 (10)	0.0024 (10)
C58A	0.029 (5)	0.021 (5)	0.036 (5)	−0.003 (4)	−0.002 (5)	0.003 (4)
C59	0.0193 (10)	0.0315 (12)	0.0282 (11)	−0.0073 (9)	0.0002 (9)	−0.0046 (9)
C60	0.0226 (12)	0.0374 (14)	0.0553 (16)	−0.0046 (10)	0.0051 (11)	−0.0151 (12)
C61	0.0329 (18)	0.0360 (19)	0.052 (2)	0.0038 (14)	−0.0176 (16)	−0.0141 (17)
C62	0.0195 (16)	0.044 (2)	0.050 (2)	0.0068 (14)	−0.0089 (16)	−0.030 (2)
C61A	0.023 (4)	0.039 (4)	0.052 (5)	−0.002 (3)	−0.007 (4)	−0.001 (4)
C62A	0.035 (4)	0.046 (5)	0.068 (5)	0.006 (4)	−0.003 (5)	−0.007 (5)
C63	0.0349 (14)	0.0359 (14)	0.0436 (15)	0.0008 (11)	0.0030 (11)	−0.0090 (11)
C64	0.0337 (15)	0.0304 (15)	0.0283 (15)	0.0009 (12)	−0.0031 (12)	0.0046 (12)
C63A	0.0349 (14)	0.0359 (14)	0.0436 (15)	0.0008 (11)	0.0030 (11)	−0.0090 (11)
C64A	0.039 (6)	0.039 (6)	0.031 (5)	0.002 (5)	0.002 (5)	−0.004 (5)
C65	0.0239 (12)	0.0337 (13)	0.0403 (14)	0.0049 (10)	−0.0023 (10)	−0.0087 (11)
C66	0.0309 (13)	0.0315 (13)	0.0385 (13)	0.0023 (10)	0.0015 (10)	−0.0156 (11)
C67	0.0315 (13)	0.0591 (18)	0.0367 (14)	0.0066 (12)	−0.0092 (11)	−0.0300 (13)
C68	0.0211 (11)	0.0633 (17)	0.0189 (11)	−0.0068 (11)	0.0020 (9)	−0.0119 (11)
C69	0.0414 (14)	0.0331 (13)	0.0213 (11)	−0.0182 (11)	−0.0102 (10)	0.0034 (9)
C70	0.0406 (14)	0.0227 (11)	0.0361 (13)	−0.0025 (10)	−0.0213 (11)	−0.0004 (10)
C71	0.0317 (13)	0.0216 (11)	0.0448 (14)	0.0006 (9)	−0.0050 (11)	−0.0056 (10)
C72	0.0337 (13)	0.0225 (12)	0.0386 (13)	−0.0034 (10)	−0.0044 (10)	0.0047 (10)
C73	0.0279 (12)	0.0272 (12)	0.0371 (13)	−0.0043 (9)	0.0030 (10)	−0.0042 (10)
C74	0.0239 (11)	0.0247 (12)	0.0447 (14)	−0.0065 (9)	−0.0075 (10)	−0.0104 (10)
C75	0.0199 (11)	0.0238 (11)	0.0423 (13)	−0.0003 (8)	−0.0136 (9)	−0.0072 (10)
C76	0.0194 (11)	0.0218 (11)	0.0440 (14)	0.0022 (8)	−0.0109 (9)	−0.0097 (10)
C77	0.0304 (12)	0.0287 (12)	0.0369 (13)	0.0033 (10)	−0.0116 (10)	−0.0011 (10)
C78	0.0231 (11)	0.0331 (13)	0.0370 (13)	−0.0003 (9)	−0.0098 (10)	−0.0067 (10)
C79	0.0284 (12)	0.0287 (12)	0.0299 (12)	−0.0096 (9)	−0.0108 (9)	−0.0054 (9)
C80	0.0342 (12)	0.0300 (12)	0.0231 (11)	−0.0074 (10)	−0.0076 (9)	−0.0096 (9)
C81	0.0226 (11)	0.0229 (11)	0.0315 (12)	−0.0023 (8)	−0.0027 (9)	−0.0124 (9)
C82	0.0286 (12)	0.0204 (11)	0.0370 (13)	−0.0039 (9)	−0.0090 (10)	−0.0074 (9)
O13	0.0622 (17)	0.0743 (18)	0.162 (3)	0.0238 (13)	−0.0697 (19)	−0.062 (2)
C83	0.060 (2)	0.058 (2)	0.080 (3)	0.0058 (17)	0.017 (2)	−0.0003 (19)
C84	0.066 (2)	0.120 (4)	0.053 (2)	−0.028 (2)	−0.0221 (18)	−0.004 (2)
C85	0.044 (2)	0.107 (3)	0.086 (3)	−0.015 (2)	−0.0024 (19)	−0.019 (2)
C86	0.127 (4)	0.090 (3)	0.068 (3)	0.056 (3)	−0.047 (3)	−0.041 (2)
O14	0.096 (3)	0.069 (3)	0.041 (2)	−0.008 (2)	−0.004 (2)	0.0043 (18)
C87	0.099 (5)	0.050 (4)	0.041 (4)	−0.011 (4)	−0.017 (4)	0.001 (3)
C88	0.076 (5)	0.062 (7)	0.045 (5)	−0.047 (4)	−0.015 (4)	0.012 (4)
C89	0.069 (4)	0.083 (7)	0.055 (5)	−0.041 (5)	−0.023 (3)	0.028 (5)
C90	0.101 (5)	0.054 (3)	0.046 (3)	−0.016 (3)	−0.006 (3)	−0.004 (2)
O14A	0.131 (7)	0.047 (4)	0.069 (4)	0.015 (4)	−0.058 (4)	−0.022 (3)
C87A	0.109 (10)	0.104 (10)	0.086 (8)	0.031 (8)	−0.054 (7)	−0.048 (7)
C88A	0.153 (12)	0.021 (5)	0.066 (8)	−0.023 (5)	−0.061 (8)	−0.003 (5)
C89A	0.135 (13)	0.031 (6)	0.058 (9)	−0.023 (7)	−0.012 (8)	0.002 (6)
C90A	0.093 (10)	0.063 (7)	0.079 (9)	0.012 (7)	−0.035 (8)	−0.033 (7)

### Geometric parameters (Å, º)

**Table:**

Fe1—N1	1.9499 (16)	C45—C46	1.392 (3)
Fe1—N3	1.9601 (16)	C45—H45	0.9500
Fe1—N2	1.9699 (16)	C46—H46	0.9500
Fe1—N4	1.9757 (16)	C47—C48	1.499 (3)
Fe1—C27	1.988 (2)	C47—H47A	0.9900
Fe1—C28	1.991 (2)	C47—H47B	0.9900
K1—O3	2.6918 (15)	C48—H48A	0.9900
K1—O1	2.7581 (14)	C48—H48B	0.9900
K1—O4	2.7780 (16)	C49—C50	1.498 (3)
K1—O2	2.8205 (16)	C49—H49A	0.9900
K1—N5	2.8735 (19)	C49—H49B	0.9900
K1—N10	3.015 (2)	C50—H50A	0.9900
K1—N9	3.0475 (18)	C50—H50B	0.9900
K1—O5	3.142 (2)	C51—C52	1.520 (3)
K1—O6A	3.150 (13)	C51—H51A	0.9900
K1—O6	3.184 (4)	C51—H51B	0.9900
K2—O9	2.6911 (16)	C52—H52A	0.9900
K2—O8	2.7978 (15)	C52—H52B	0.9900
K2—O12	2.8144 (16)	C51A—C52A	1.481 (7)
K2—O10	2.8282 (15)	C51A—H51C	0.9900
K2—N8	2.9787 (19)	C51A—H51D	0.9900
K2—O11	3.0001 (15)	C52A—H52C	0.9900
K2—N6	3.0257 (18)	C52A—H52D	0.9900
K2—O7	3.0629 (16)	C53—C54	1.511 (3)
K2—N7	3.0686 (18)	C53—H53A	0.9900
Cl1—C38	1.745 (2)	C53—H53B	0.9900
Cl2—C24	1.746 (2)	C54—H54A	0.9900
Cl3—C44	1.746 (2)	C54—H54B	0.9900
Cl4—C33	1.747 (2)	C55—C56	1.500 (4)
O1—C49	1.414 (3)	C55—H55A	0.9900
O1—C48	1.435 (2)	C55—H55B	0.9900
O2—C50	1.418 (3)	C56—H56A	0.9900
O2—C51A	1.423 (3)	C56—H56B	0.9900
O2—C51	1.423 (3)	C57—C58	1.484 (4)
O3—C56	1.415 (3)	C57—H57A	0.9900
O3—C57A	1.424 (3)	C57—H57B	0.9900
O3—C57	1.424 (3)	C58—H58A	0.9900
O4—C54	1.423 (3)	C58—H58B	0.9900
O4—C55	1.428 (3)	C57A—C58A	1.511 (7)
O5—C61	1.411 (4)	C57A—H57C	0.9900
O5—C60	1.428 (4)	C57A—H57D	0.9900
O5—C61A	1.542 (7)	C58A—H58C	0.9900
O6—C63	1.441 (4)	C58A—H58D	0.9900
O6—C62	1.471 (4)	C59—C60	1.499 (3)
O6A—C63A	1.419 (7)	C59—H59A	0.9900
O6A—C62A	1.461 (8)	C59—H59B	0.9900
O7—C73	1.405 (3)	C60—H60A	0.9900
O7—C72	1.425 (3)	C60—H60B	0.9900
O8—C81	1.421 (3)	C61—C62	1.508 (5)
O8—C80	1.427 (3)	C61—H61A	0.9900
O9—C66	1.406 (3)	C61—H61B	0.9900
O9—C67	1.418 (3)	C62—H62A	0.9900
O10—C69	1.421 (3)	C62—H62B	0.9900
O10—C68	1.430 (3)	C61A—C62A	1.503 (9)
O11—C74	1.423 (3)	C61A—H61C	0.9900
O11—C75	1.424 (3)	C61A—H61D	0.9900
O12—C78	1.426 (3)	C62A—H62C	0.9900
O12—C79	1.432 (3)	C62A—H62D	0.9900
N1—C1	1.374 (2)	C63—C64	1.502 (3)
N1—C4	1.375 (2)	C63—H63A	0.9900
N2—C9	1.376 (2)	C63—H63B	0.9900
N2—C6	1.379 (2)	C64—H64A	0.9900
N3—C14	1.375 (3)	C64—H64B	0.9900
N3—C11	1.376 (2)	C63A—C64A	1.530 (8)
N4—C16	1.376 (3)	C63A—H63C	0.9900
N4—C19	1.382 (2)	C63A—H63D	0.9900
N5—C27	1.160 (3)	C64A—H64C	0.9900
N6—C28	1.159 (3)	C64A—H64D	0.9900
N7—C76	1.464 (3)	C65—C66	1.508 (4)
N7—C70	1.473 (3)	C65—H65A	0.9900
N7—C82	1.477 (3)	C65—H65B	0.9900
N8—C77	1.469 (3)	C66—H66A	0.9900
N8—C71	1.469 (3)	C66—H66B	0.9900
N8—C65	1.469 (3)	C67—C68	1.489 (4)
N9—C53	1.464 (3)	C67—H67A	0.9900
N9—C47	1.471 (3)	C67—H67B	0.9900
N9—C59	1.475 (3)	C68—H68A	0.9900
N10—C52	1.442 (3)	C68—H68B	0.9900
N10—C58A	1.447 (7)	C69—C70	1.506 (4)
N10—C58	1.475 (3)	C69—H69A	0.9900
N10—C64A	1.480 (7)	C69—H69B	0.9900
N10—C64	1.480 (3)	C70—H70A	0.9900
N10—C52A	1.481 (7)	C70—H70B	0.9900
C1—C20	1.398 (3)	C71—C72	1.498 (3)
C1—C2	1.439 (3)	C71—H71A	0.9900
C2—C3	1.351 (3)	C71—H71B	0.9900
C2—H2	0.9500	C72—H72A	0.9900
C3—C4	1.445 (3)	C72—H72B	0.9900
C3—H3	0.9500	C73—C74	1.507 (3)
C4—C5	1.397 (3)	C73—H73A	0.9900
C5—C6	1.400 (3)	C73—H73B	0.9900
C5—C21	1.493 (3)	C74—H74A	0.9900
C6—C7	1.440 (3)	C74—H74B	0.9900
C7—C8	1.351 (3)	C75—C76	1.510 (3)
C7—H7	0.9500	C75—H75A	0.9900
C8—C9	1.442 (3)	C75—H75B	0.9900
C8—H8	0.9500	C76—H76A	0.9900
C9—C10	1.401 (3)	C76—H76B	0.9900
C10—C11	1.396 (3)	C77—C78	1.511 (3)
C10—C41	1.494 (3)	C77—H77A	0.9900
C11—C12	1.444 (3)	C77—H77B	0.9900
C12—C13	1.346 (3)	C78—H78A	0.9900
C12—H12	0.9500	C78—H78B	0.9900
C13—C14	1.444 (3)	C79—C80	1.490 (3)
C13—H13	0.9500	C79—H79A	0.9900
C14—C15	1.401 (3)	C79—H79B	0.9900
C15—C16	1.399 (3)	C80—H80A	0.9900
C15—C35	1.493 (3)	C80—H80B	0.9900
C16—C17	1.447 (3)	C81—C82	1.500 (3)
C17—C18	1.351 (3)	C81—H81A	0.9900
C17—H17	0.9500	C81—H81B	0.9900
C18—C19	1.443 (3)	C82—H82A	0.9900
C18—H18	0.9500	C82—H82B	0.9900
C19—C20	1.396 (3)	O13—C83	1.377 (5)
C20—C29	1.499 (3)	O13—C86	1.407 (5)
C21—C22	1.391 (3)	C83—C84	1.498 (5)
C21—C26	1.404 (3)	C83—H83A	0.9900
C22—C23	1.393 (3)	C83—H83B	0.9900
C22—H22	0.9500	C84—C85	1.462 (6)
C23—C24	1.372 (3)	C84—H84A	0.9900
C23—H23	0.9500	C84—H84B	0.9900
C24—C25	1.380 (3)	C85—C86	1.440 (5)
C25—C26	1.388 (3)	C85—H85A	0.9900
C25—H25	0.9500	C85—H85B	0.9900
C26—H26	0.9500	C86—H86A	0.9900
C29—C31	1.387 (3)	C86—H86B	0.9900
C29—C30	1.392 (3)	O14—C87	1.415 (7)
C30—C34	1.389 (3)	O14—C90	1.417 (7)
C30—H30	0.9500	C87—C88	1.515 (13)
C31—C32	1.391 (3)	C87—H87A	0.9900
C31—H31	0.9500	C87—H87B	0.9900
C32—C33	1.379 (3)	C88—C89	1.527 (17)
C32—H32	0.9500	C88—H88A	0.9900
C33—C34	1.381 (3)	C88—H88B	0.9900
C34—H34	0.9500	C89—C90	1.499 (11)
C35—C36	1.392 (3)	C89—H89A	0.9900
C35—C40	1.396 (3)	C89—H89B	0.9900
C36—C37	1.384 (3)	C90—H90A	0.9900
C36—H36	0.9500	C90—H90B	0.9900
C37—C38	1.380 (3)	O14A—C87A	1.406 (8)
C37—H37	0.9500	O14A—C90A	1.423 (16)
C38—C39	1.381 (3)	C87A—C88A	1.570 (17)
C39—C40	1.392 (3)	C87A—H87C	0.9900
C39—H39	0.9500	C87A—H87D	0.9900
C40—H40	0.9500	C88A—C89A	1.58 (3)
C41—C46	1.392 (3)	C88A—H88C	0.9900
C41—C42	1.404 (3)	C88A—H88D	0.9900
C42—C43	1.387 (3)	C89A—C90A	1.45 (3)
C42—H42	0.9500	C89A—H89C	0.9900
C43—C44	1.379 (3)	C89A—H89D	0.9900
C43—H43	0.9500	C90A—H90C	0.9900
C44—C45	1.384 (3)	C90A—H90D	0.9900
			
N1—Fe1—N3	178.64 (7)	H48A—C48—H48B	108.4
N1—Fe1—N2	89.69 (7)	O1—C49—C50	109.03 (18)
N3—Fe1—N2	89.82 (7)	O1—C49—H49A	109.9
N1—Fe1—N4	89.91 (7)	C50—C49—H49A	109.9
N3—Fe1—N4	90.60 (7)	O1—C49—H49B	109.9
N2—Fe1—N4	179.28 (7)	C50—C49—H49B	109.9
N1—Fe1—C27	89.86 (7)	H49A—C49—H49B	108.3
N3—Fe1—C27	88.88 (7)	O2—C50—C49	109.05 (18)
N2—Fe1—C27	90.91 (7)	O2—C50—H50A	109.9
N4—Fe1—C27	89.69 (7)	C49—C50—H50A	109.9
N1—Fe1—C28	90.01 (7)	O2—C50—H50B	109.9
N3—Fe1—C28	91.25 (7)	C49—C50—H50B	109.9
N2—Fe1—C28	90.12 (7)	H50A—C50—H50B	108.3
N4—Fe1—C28	89.29 (7)	O2—C51—C52	107.9 (2)
C27—Fe1—C28	178.96 (8)	O2—C51—H51A	110.1
O3—K1—O1	169.37 (5)	C52—C51—H51A	110.1
O3—K1—O4	63.42 (5)	O2—C51—H51B	110.1
O1—K1—O4	115.80 (5)	C52—C51—H51B	110.1
O3—K1—O2	117.87 (5)	H51A—C51—H51B	108.4
O1—K1—O2	61.06 (4)	N10—C52—C51	114.0 (2)
O4—K1—O2	170.83 (5)	N10—C52—H52A	108.8
O3—K1—N5	74.53 (5)	C51—C52—H52A	108.8
O1—K1—N5	94.87 (5)	N10—C52—H52B	108.8
O4—K1—N5	85.35 (5)	C51—C52—H52B	108.8
O2—K1—N5	86.36 (5)	H52A—C52—H52B	107.7
O3—K1—N10	59.74 (5)	O2—C51A—C52A	121.3 (4)
O1—K1—N10	120.75 (5)	O2—C51A—H51C	107.0
O4—K1—N10	123.02 (5)	C52A—C51A—H51C	107.0
O2—K1—N10	59.73 (5)	O2—C51A—H51D	107.0
N5—K1—N10	83.63 (5)	C52A—C51A—H51D	107.0
O3—K1—N9	123.55 (5)	H51C—C51A—H51D	106.7
O1—K1—N9	58.91 (5)	C51A—C52A—N10	114.0 (6)
O4—K1—N9	61.14 (5)	C51A—C52A—H52C	108.8
O2—K1—N9	118.56 (5)	N10—C52A—H52C	108.8
N5—K1—N9	110.15 (5)	C51A—C52A—H52D	108.8
N10—K1—N9	166.19 (5)	N10—C52A—H52D	108.8
O3—K1—O5	98.09 (6)	H52C—C52A—H52D	107.6
O1—K1—O5	91.61 (5)	N9—C53—C54	112.95 (19)
O4—K1—O5	73.84 (6)	N9—C53—H53A	109.0
O2—K1—O5	114.16 (6)	C54—C53—H53A	109.0
N5—K1—O5	158.98 (6)	N9—C53—H53B	109.0
N10—K1—O5	110.02 (5)	C54—C53—H53B	109.0
N9—K1—O5	57.12 (5)	H53A—C53—H53B	107.8
O3—K1—O6A	81.1 (2)	O4—C54—C53	108.45 (18)
O1—K1—O6A	108.4 (2)	O4—C54—H54A	110.0
O4—K1—O6A	109.8 (2)	C53—C54—H54A	110.0
O2—K1—O6A	79.2 (2)	O4—C54—H54B	110.0
N5—K1—O6A	141.45 (15)	C53—C54—H54B	110.0
N10—K1—O6A	58.19 (14)	H54A—C54—H54B	108.4
N9—K1—O6A	108.16 (14)	O4—C55—C56	109.23 (19)
O5—K1—O6A	52.86 (15)	O4—C55—H55A	109.8
O3—K1—O6	70.45 (8)	C56—C55—H55A	109.8
O1—K1—O6	119.31 (8)	O4—C55—H55B	109.8
O4—K1—O6	101.19 (7)	C56—C55—H55B	109.8
O2—K1—O6	87.53 (7)	H55A—C55—H55B	108.3
N5—K1—O6	136.28 (7)	O3—C56—C55	108.85 (19)
N10—K1—O6	56.38 (6)	O3—C56—H56A	109.9
N9—K1—O6	110.73 (6)	C55—C56—H56A	109.9
O5—K1—O6	53.68 (6)	O3—C56—H56B	109.9
O9—K2—O8	153.42 (5)	C55—C56—H56B	109.9
O9—K2—O12	116.80 (5)	H56A—C56—H56B	108.3
O8—K2—O12	61.49 (4)	O3—C57—C58	113.84 (19)
O9—K2—O10	61.52 (5)	O3—C57—H57A	108.8
O8—K2—O10	109.76 (5)	C58—C57—H57A	108.8
O12—K2—O10	159.00 (5)	O3—C57—H57B	108.8
O9—K2—N8	59.00 (5)	C58—C57—H57B	108.8
O8—K2—N8	124.36 (5)	H57A—C57—H57B	107.7
O12—K2—N8	63.06 (5)	N10—C58—C57	116.6 (2)
O10—K2—N8	120.46 (5)	N10—C58—H58A	108.1
O9—K2—O11	108.27 (5)	C57—C58—H58A	108.1
O8—K2—O11	94.66 (4)	N10—C58—H58B	108.1
O12—K2—O11	115.43 (5)	C57—C58—H58B	108.1
O10—K2—O11	83.25 (4)	H58A—C58—H58B	107.3
N8—K2—O11	112.65 (5)	O3—C57A—C58A	109.5 (5)
O9—K2—N6	72.08 (5)	O3—C57A—H57C	109.8
O8—K2—N6	81.78 (5)	C58A—C57A—H57C	109.8
O12—K2—N6	80.09 (5)	O3—C57A—H57D	109.8
O10—K2—N6	79.70 (5)	C58A—C57A—H57D	109.8
N8—K2—N6	84.92 (5)	H57C—C57A—H57D	108.2
O11—K2—N6	160.18 (5)	N10—C58A—C57A	116.6 (6)
O9—K2—O7	73.57 (5)	N10—C58A—H58C	108.1
O8—K2—O7	132.12 (5)	C57A—C58A—H58C	108.1
O12—K2—O7	95.10 (5)	N10—C58A—H58D	108.1
O10—K2—O7	103.78 (5)	C57A—C58A—H58D	108.1
N8—K2—O7	57.04 (5)	H58C—C58A—H58D	107.3
O11—K2—O7	56.21 (4)	N9—C59—C60	113.83 (18)
N6—K2—O7	138.27 (5)	N9—C59—H59A	108.8
O9—K2—N7	121.43 (5)	C60—C59—H59A	108.8
O8—K2—N7	59.80 (5)	N9—C59—H59B	108.8
O12—K2—N7	119.75 (5)	C60—C59—H59B	108.8
O10—K2—N7	60.34 (5)	H59A—C59—H59B	107.7
N8—K2—N7	171.28 (5)	O5—C60—C59	108.1 (2)
O11—K2—N7	58.64 (4)	O5—C60—H60A	110.1
N6—K2—N7	103.60 (5)	C59—C60—H60A	110.1
O7—K2—N7	114.28 (5)	O5—C60—H60B	110.1
C49—O1—C48	112.15 (16)	C59—C60—H60B	110.1
C49—O1—K1	115.66 (12)	H60A—C60—H60B	108.4
C48—O1—K1	125.40 (12)	O5—C61—C62	109.2 (3)
C50—O2—C51A	111.64 (17)	O5—C61—H61A	109.8
C50—O2—C51	111.64 (17)	C62—C61—H61A	109.8
C50—O2—K1	111.50 (13)	O5—C61—H61B	109.8
C51A—O2—K1	108.62 (14)	C62—C61—H61B	109.8
C51—O2—K1	108.62 (14)	H61A—C61—H61B	108.3
C56—O3—C57A	112.17 (17)	O6—C62—C61	106.0 (3)
C56—O3—C57	112.17 (17)	O6—C62—H62A	110.5
C56—O3—K1	113.25 (13)	C61—C62—H62A	110.5
C57A—O3—K1	118.94 (13)	O6—C62—H62B	110.5
C57—O3—K1	118.94 (13)	C61—C62—H62B	110.5
C54—O4—C55	112.63 (17)	H62A—C62—H62B	108.7
C54—O4—K1	116.09 (12)	C62A—C61A—O5	108.3 (8)
C55—O4—K1	109.03 (13)	C62A—C61A—H61C	110.0
C61—O5—C60	121.2 (3)	O5—C61A—H61C	110.0
C60—O5—C61A	92.1 (4)	C62A—C61A—H61D	110.0
C61—O5—K1	120.05 (19)	O5—C61A—H61D	110.0
C60—O5—K1	93.93 (14)	H61C—C61A—H61D	108.4
C61A—O5—K1	105.0 (4)	O6A—C62A—C61A	88.2 (8)
C63—O6—C62	108.7 (3)	O6A—C62A—H62C	114.0
C63—O6—K1	110.3 (2)	C61A—C62A—H62C	114.0
C62—O6—K1	108.1 (3)	O6A—C62A—H62D	114.0
C63A—O6A—C62A	116.1 (9)	C61A—C62A—H62D	114.0
C63A—O6A—K1	112.8 (6)	H62C—C62A—H62D	111.2
C62A—O6A—K1	120.6 (8)	O6—C63—C64	105.6 (2)
C73—O7—C72	111.71 (18)	O6—C63—H63A	110.6
C73—O7—K2	115.73 (13)	C64—C63—H63A	110.6
C72—O7—K2	113.57 (13)	O6—C63—H63B	110.6
C81—O8—C80	111.81 (16)	C64—C63—H63B	110.6
C81—O8—K2	120.84 (12)	H63A—C63—H63B	108.7
C80—O8—K2	115.24 (12)	N10—C64—C63	110.9 (2)
C66—O9—C67	114.32 (18)	N10—C64—H64A	109.5
C66—O9—K2	125.25 (14)	C63—C64—H64A	109.5
C67—O9—K2	118.81 (14)	N10—C64—H64B	109.5
C69—O10—C68	111.55 (18)	C63—C64—H64B	109.5
C69—O10—K2	103.29 (12)	H64A—C64—H64B	108.1
C68—O10—K2	106.74 (12)	O6A—C63A—C64A	118.5 (8)
C74—O11—C75	110.36 (16)	O6A—C63A—H63C	107.7
C74—O11—K2	114.66 (12)	C64A—C63A—H63C	107.7
C75—O11—K2	114.77 (12)	O6A—C63A—H63D	107.7
C78—O12—C79	109.92 (17)	C64A—C63A—H63D	107.7
C78—O12—K2	106.31 (13)	H63C—C63A—H63D	107.1
C79—O12—K2	108.32 (12)	N10—C64A—C63A	109.3 (6)
C1—N1—C4	106.16 (15)	N10—C64A—H64C	109.8
C1—N1—Fe1	126.97 (13)	C63A—C64A—H64C	109.8
C4—N1—Fe1	126.85 (13)	N10—C64A—H64D	109.8
C9—N2—C6	106.14 (16)	C63A—C64A—H64D	109.8
C9—N2—Fe1	126.60 (13)	H64C—C64A—H64D	108.3
C6—N2—Fe1	127.26 (13)	N8—C65—C66	114.7 (2)
C14—N3—C11	106.31 (16)	N8—C65—H65A	108.6
C14—N3—Fe1	126.09 (13)	C66—C65—H65A	108.6
C11—N3—Fe1	127.59 (14)	N8—C65—H65B	108.6
C16—N4—C19	105.76 (16)	C66—C65—H65B	108.6
C16—N4—Fe1	127.08 (13)	H65A—C65—H65B	107.6
C19—N4—Fe1	127.15 (13)	O9—C66—C65	109.21 (19)
C27—N5—K1	153.75 (16)	O9—C66—H66A	109.8
C28—N6—K2	152.75 (15)	C65—C66—H66A	109.8
C76—N7—C70	111.77 (18)	O9—C66—H66B	109.8
C76—N7—C82	110.50 (18)	C65—C66—H66B	109.8
C70—N7—C82	109.25 (18)	H66A—C66—H66B	108.3
C76—N7—K2	111.34 (12)	O9—C67—C68	109.0 (2)
C70—N7—K2	107.29 (13)	O9—C67—H67A	109.9
C82—N7—K2	106.49 (12)	C68—C67—H67A	109.9
C77—N8—C71	110.43 (19)	O9—C67—H67B	109.9
C77—N8—C65	110.15 (19)	C68—C67—H67B	109.9
C71—N8—C65	111.37 (19)	H67A—C67—H67B	108.3
C77—N8—K2	107.20 (13)	O10—C68—C67	109.22 (19)
C71—N8—K2	117.21 (13)	O10—C68—H68A	109.8
C65—N8—K2	99.93 (13)	C67—C68—H68A	109.8
C53—N9—C47	110.26 (17)	O10—C68—H68B	109.8
C53—N9—C59	111.96 (17)	C67—C68—H68B	109.8
C47—N9—C59	111.10 (17)	H68A—C68—H68B	108.3
C53—N9—K1	105.89 (12)	O10—C69—C70	108.17 (18)
C47—N9—K1	105.64 (12)	O10—C69—H69A	110.1
C59—N9—K1	111.68 (13)	C70—C69—H69A	110.1
C52—N10—C58	110.8 (2)	O10—C69—H69B	110.1
C58A—N10—C64A	116.3 (7)	C70—C69—H69B	110.1
C52—N10—C64	111.2 (2)	H69A—C69—H69B	108.4
C58—N10—C64	110.80 (19)	N7—C70—C69	112.64 (18)
C58A—N10—C52A	107.8 (7)	N7—C70—H70A	109.1
C64A—N10—C52A	110.9 (6)	C69—C70—H70A	109.1
C52—N10—K1	110.87 (14)	N7—C70—H70B	109.1
C58A—N10—K1	111.3 (4)	C69—C70—H70B	109.1
C58—N10—K1	96.48 (14)	H70A—C70—H70B	107.8
C64A—N10—K1	113.0 (5)	N8—C71—C72	112.3 (2)
C64—N10—K1	115.83 (16)	N8—C71—H71A	109.1
C52A—N10—K1	95.4 (5)	C72—C71—H71A	109.1
N1—C1—C20	125.21 (18)	N8—C71—H71B	109.1
N1—C1—C2	109.99 (17)	C72—C71—H71B	109.1
C20—C1—C2	124.18 (18)	H71A—C71—H71B	107.9
C3—C2—C1	107.02 (17)	O7—C72—C71	108.19 (19)
C3—C2—H2	126.5	O7—C72—H72A	110.1
C1—C2—H2	126.5	C71—C72—H72A	110.1
C2—C3—C4	107.07 (17)	O7—C72—H72B	110.1
C2—C3—H3	126.5	C71—C72—H72B	110.1
C4—C3—H3	126.5	H72A—C72—H72B	108.4
N1—C4—C5	125.03 (17)	O7—C73—C74	108.62 (19)
N1—C4—C3	109.68 (16)	O7—C73—H73A	110.0
C5—C4—C3	124.60 (18)	C74—C73—H73A	110.0
C4—C5—C6	122.88 (18)	O7—C73—H73B	110.0
C4—C5—C21	116.87 (17)	C74—C73—H73B	110.0
C6—C5—C21	120.17 (17)	H73A—C73—H73B	108.3
N2—C6—C5	124.10 (17)	O11—C74—C73	109.47 (18)
N2—C6—C7	109.83 (16)	O11—C74—H74A	109.8
C5—C6—C7	125.98 (18)	C73—C74—H74A	109.8
C8—C7—C6	107.04 (18)	O11—C74—H74B	109.8
C8—C7—H7	126.5	C73—C74—H74B	109.8
C6—C7—H7	126.5	H74A—C74—H74B	108.2
C7—C8—C9	107.25 (17)	O11—C75—C76	110.00 (17)
C7—C8—H8	126.4	O11—C75—H75A	109.7
C9—C8—H8	126.4	C76—C75—H75A	109.7
N2—C9—C10	125.01 (18)	O11—C75—H75B	109.7
N2—C9—C8	109.68 (17)	C76—C75—H75B	109.7
C10—C9—C8	124.66 (18)	H75A—C75—H75B	108.2
C11—C10—C9	123.05 (18)	N7—C76—C75	113.28 (18)
C11—C10—C41	119.44 (17)	N7—C76—H76A	108.9
C9—C10—C41	117.48 (18)	C75—C76—H76A	108.9
N3—C11—C10	124.54 (18)	N7—C76—H76B	108.9
N3—C11—C12	109.59 (17)	C75—C76—H76B	108.9
C10—C11—C12	125.82 (18)	H76A—C76—H76B	107.7
C13—C12—C11	107.22 (18)	N8—C77—C78	114.13 (19)
C13—C12—H12	126.4	N8—C77—H77A	108.7
C11—C12—H12	126.4	C78—C77—H77A	108.7
C12—C13—C14	107.19 (18)	N8—C77—H77B	108.7
C12—C13—H13	126.4	C78—C77—H77B	108.7
C14—C13—H13	126.4	H77A—C77—H77B	107.6
N3—C14—C15	125.66 (18)	O12—C78—C77	110.04 (19)
N3—C14—C13	109.61 (17)	O12—C78—H78A	109.7
C15—C14—C13	124.32 (18)	C77—C78—H78A	109.7
C16—C15—C14	123.63 (18)	O12—C78—H78B	109.7
C16—C15—C35	120.43 (18)	C77—C78—H78B	109.7
C14—C15—C35	115.81 (17)	H78A—C78—H78B	108.2
N4—C16—C15	123.94 (18)	O12—C79—C80	110.17 (18)
N4—C16—C17	110.09 (17)	O12—C79—H79A	109.6
C15—C16—C17	125.73 (18)	C80—C79—H79A	109.6
C18—C17—C16	106.93 (18)	O12—C79—H79B	109.6
C18—C17—H17	126.5	C80—C79—H79B	109.6
C16—C17—H17	126.5	H79A—C79—H79B	108.1
C17—C18—C19	107.12 (17)	O8—C80—C79	107.73 (18)
C17—C18—H18	126.4	O8—C80—H80A	110.2
C19—C18—H18	126.4	C79—C80—H80A	110.2
N4—C19—C20	123.94 (17)	O8—C80—H80B	110.2
N4—C19—C18	109.96 (17)	C79—C80—H80B	110.2
C20—C19—C18	125.71 (18)	H80A—C80—H80B	108.5
C19—C20—C1	123.29 (18)	O8—C81—C82	108.08 (17)
C19—C20—C29	120.92 (17)	O8—C81—H81A	110.1
C1—C20—C29	115.51 (17)	C82—C81—H81A	110.1
C22—C21—C26	118.21 (18)	O8—C81—H81B	110.1
C22—C21—C5	123.37 (18)	C82—C81—H81B	110.1
C26—C21—C5	118.42 (17)	H81A—C81—H81B	108.4
C21—C22—C23	120.6 (2)	N7—C82—C81	114.31 (18)
C21—C22—H22	119.7	N7—C82—H82A	108.7
C23—C22—H22	119.7	C81—C82—H82A	108.7
C24—C23—C22	119.7 (2)	N7—C82—H82B	108.7
C24—C23—H23	120.1	C81—C82—H82B	108.7
C22—C23—H23	120.1	H82A—C82—H82B	107.6
C23—C24—C25	121.4 (2)	C83—O13—C86	108.8 (3)
C23—C24—Cl2	119.33 (18)	O13—C83—C84	107.6 (3)
C25—C24—Cl2	119.26 (18)	O13—C83—H83A	110.2
C24—C25—C26	118.8 (2)	C84—C83—H83A	110.2
C24—C25—H25	120.6	O13—C83—H83B	110.2
C26—C25—H25	120.6	C84—C83—H83B	110.2
C25—C26—C21	121.28 (19)	H83A—C83—H83B	108.5
C25—C26—H26	119.4	C85—C84—C83	104.1 (3)
C21—C26—H26	119.4	C85—C84—H84A	110.9
N5—C27—Fe1	178.62 (18)	C83—C84—H84A	110.9
N6—C28—Fe1	178.83 (19)	C85—C84—H84B	110.9
C31—C29—C30	118.11 (19)	C83—C84—H84B	110.9
C31—C29—C20	122.49 (18)	H84A—C84—H84B	109.0
C30—C29—C20	119.10 (18)	C86—C85—C84	104.6 (3)
C34—C30—C29	121.9 (2)	C86—C85—H85A	110.8
C34—C30—H30	119.0	C84—C85—H85A	110.8
C29—C30—H30	119.0	C86—C85—H85B	110.8
C29—C31—C32	121.0 (2)	C84—C85—H85B	110.8
C29—C31—H31	119.5	H85A—C85—H85B	108.9
C32—C31—H31	119.5	O13—C86—C85	106.6 (3)
C33—C32—C31	119.3 (2)	O13—C86—H86A	110.4
C33—C32—H32	120.3	C85—C86—H86A	110.4
C31—C32—H32	120.3	O13—C86—H86B	110.4
C32—C33—C34	121.4 (2)	C85—C86—H86B	110.4
C32—C33—Cl4	119.34 (18)	H86A—C86—H86B	108.6
C34—C33—Cl4	119.24 (18)	C87—O14—C90	108.4 (5)
C33—C34—C30	118.3 (2)	O14—C87—C88	108.3 (7)
C33—C34—H34	120.9	O14—C87—H87A	110.0
C30—C34—H34	120.9	C88—C87—H87A	110.0
C36—C35—C40	118.37 (19)	O14—C87—H87B	110.0
C36—C35—C15	118.52 (18)	C88—C87—H87B	110.0
C40—C35—C15	122.91 (18)	H87A—C87—H87B	108.4
C37—C36—C35	121.5 (2)	C87—C88—C89	98.5 (8)
C37—C36—H36	119.3	C87—C88—H88A	112.1
C35—C36—H36	119.3	C89—C88—H88A	112.1
C38—C37—C36	118.8 (2)	C87—C88—H88B	112.1
C38—C37—H37	120.6	C89—C88—H88B	112.1
C36—C37—H37	120.6	H88A—C88—H88B	109.7
C37—C38—C39	121.6 (2)	C90—C89—C88	106.8 (8)
C37—C38—Cl1	118.85 (18)	C90—C89—H89A	110.4
C39—C38—Cl1	119.59 (18)	C88—C89—H89A	110.4
C38—C39—C40	119.1 (2)	C90—C89—H89B	110.4
C38—C39—H39	120.5	C88—C89—H89B	110.4
C40—C39—H39	120.5	H89A—C89—H89B	108.6
C39—C40—C35	120.7 (2)	O14—C90—C89	106.7 (6)
C39—C40—H40	119.6	O14—C90—H90A	110.4
C35—C40—H40	119.6	C89—C90—H90A	110.4
C46—C41—C42	117.76 (18)	O14—C90—H90B	110.4
C46—C41—C10	119.23 (18)	C89—C90—H90B	110.4
C42—C41—C10	123.02 (19)	H90A—C90—H90B	108.6
C43—C42—C41	121.4 (2)	C87A—O14A—C90A	107.9 (9)
C43—C42—H42	119.3	O14A—C87A—C88A	109.4 (11)
C41—C42—H42	119.3	O14A—C87A—H87C	109.8
C44—C43—C42	118.8 (2)	C88A—C87A—H87C	109.8
C44—C43—H43	120.6	O14A—C87A—H87D	109.8
C42—C43—H43	120.6	C88A—C87A—H87D	109.8
C43—C44—C45	121.90 (19)	H87C—C87A—H87D	108.2
C43—C44—Cl3	119.13 (17)	C87A—C88A—C89A	98.3 (14)
C45—C44—Cl3	118.97 (18)	C87A—C88A—H88C	112.1
C44—C45—C46	118.4 (2)	C89A—C88A—H88C	112.1
C44—C45—H45	120.8	C87A—C88A—H88D	112.1
C46—C45—H45	120.8	C89A—C88A—H88D	112.1
C41—C46—C45	121.7 (2)	H88C—C88A—H88D	109.8
C41—C46—H46	119.1	C90A—C89A—C88A	104.0 (17)
C45—C46—H46	119.1	C90A—C89A—H89C	111.0
N9—C47—C48	113.19 (18)	C88A—C89A—H89C	111.0
N9—C47—H47A	108.9	C90A—C89A—H89D	111.0
C48—C47—H47A	108.9	C88A—C89A—H89D	111.0
N9—C47—H47B	108.9	H89C—C89A—H89D	109.0
C48—C47—H47B	108.9	O14A—C90A—C89A	105.7 (14)
H47A—C47—H47B	107.8	O14A—C90A—H90C	110.6
O1—C48—C47	108.10 (17)	C89A—C90A—H90C	110.6
O1—C48—H48A	110.1	O14A—C90A—H90D	110.6
C47—C48—H48A	110.1	C89A—C90A—H90D	110.6
O1—C48—H48B	110.1	H90C—C90A—H90D	108.7
C47—C48—H48B	110.1		
			
C4—N1—C1—C20	−170.12 (19)	K1—N9—C47—C48	−55.57 (18)
Fe1—N1—C1—C20	8.3 (3)	C49—O1—C48—C47	178.70 (18)
C4—N1—C1—C2	1.2 (2)	K1—O1—C48—C47	−31.7 (2)
Fe1—N1—C1—C2	179.59 (13)	N9—C47—C48—O1	60.0 (2)
N1—C1—C2—C3	−2.6 (2)	C48—O1—C49—C50	−161.07 (18)
C20—C1—C2—C3	168.75 (19)	K1—O1—C49—C50	46.1 (2)
C1—C2—C3—C4	2.9 (2)	C51A—O2—C50—C49	173.9 (2)
C1—N1—C4—C5	−170.12 (18)	C51—O2—C50—C49	173.9 (2)
Fe1—N1—C4—C5	11.4 (3)	K1—O2—C50—C49	52.2 (2)
C1—N1—C4—C3	0.7 (2)	O1—C49—C50—O2	−66.7 (2)
Fe1—N1—C4—C3	−177.78 (13)	C50—O2—C51—C52	167.6 (2)
C2—C3—C4—N1	−2.3 (2)	K1—O2—C51—C52	−69.1 (2)
C2—C3—C4—C5	168.51 (19)	C58—N10—C52—C51	81.8 (3)
N1—C4—C5—C6	8.7 (3)	C64—N10—C52—C51	−154.5 (2)
C3—C4—C5—C6	−160.72 (19)	K1—N10—C52—C51	−24.1 (3)
N1—C4—C5—C21	−174.67 (18)	O2—C51—C52—N10	63.9 (3)
C3—C4—C5—C21	15.9 (3)	C50—O2—C51A—C52A	−147.4 (7)
C9—N2—C6—C5	175.80 (18)	K1—O2—C51A—C52A	−24.1 (7)
Fe1—N2—C6—C5	−4.5 (3)	O2—C51A—C52A—N10	−31.5 (13)
C9—N2—C6—C7	−0.9 (2)	C58A—N10—C52A—C51A	175.7 (8)
Fe1—N2—C6—C7	178.85 (13)	C64A—N10—C52A—C51A	−56.0 (12)
C4—C5—C6—N2	−12.2 (3)	K1—N10—C52A—C51A	61.2 (9)
C21—C5—C6—N2	171.30 (17)	C47—N9—C53—C54	160.00 (18)
C4—C5—C6—C7	163.93 (19)	C59—N9—C53—C54	−75.8 (2)
C21—C5—C6—C7	−12.6 (3)	K1—N9—C53—C54	46.2 (2)
N2—C6—C7—C8	2.3 (2)	C55—O4—C54—C53	−178.84 (19)
C5—C6—C7—C8	−174.31 (19)	K1—O4—C54—C53	54.5 (2)
C6—C7—C8—C9	−2.7 (2)	N9—C53—C54—O4	−70.4 (2)
C6—N2—C9—C10	170.28 (18)	C54—O4—C55—C56	178.25 (19)
Fe1—N2—C9—C10	−9.4 (3)	K1—O4—C55—C56	−51.4 (2)
C6—N2—C9—C8	−0.8 (2)	C57A—O3—C56—C55	172.47 (19)
Fe1—N2—C9—C8	179.49 (13)	C57—O3—C56—C55	172.47 (19)
C7—C8—C9—N2	2.2 (2)	K1—O3—C56—C55	−49.5 (2)
C7—C8—C9—C10	−168.87 (19)	O4—C55—C56—O3	70.1 (2)
N2—C9—C10—C11	−8.2 (3)	C56—O3—C57—C58	150.2 (2)
C8—C9—C10—C11	161.60 (19)	K1—O3—C57—C58	14.7 (3)
N2—C9—C10—C41	173.76 (17)	C52—N10—C58—C57	−175.9 (2)
C8—C9—C10—C41	−16.5 (3)	C64—N10—C58—C57	60.2 (3)
C14—N3—C11—C10	−175.65 (18)	K1—N10—C58—C57	−60.6 (2)
Fe1—N3—C11—C10	5.4 (3)	O3—C57—C58—N10	38.9 (3)
C14—N3—C11—C12	1.8 (2)	C56—O3—C57A—C58A	−167.7 (5)
Fe1—N3—C11—C12	−177.07 (13)	K1—O3—C57A—C58A	56.9 (5)
C9—C10—C11—N3	10.3 (3)	C64A—N10—C58A—C57A	152.0 (8)
C41—C10—C11—N3	−171.66 (18)	C52A—N10—C58A—C57A	−82.8 (11)
C9—C10—C11—C12	−166.78 (19)	K1—N10—C58A—C57A	20.6 (11)
C41—C10—C11—C12	11.2 (3)	O3—C57A—C58A—N10	−49.3 (11)
N3—C11—C12—C13	−2.9 (2)	C53—N9—C59—C60	128.1 (2)
C10—C11—C12—C13	174.53 (19)	C47—N9—C59—C60	−108.1 (2)
C11—C12—C13—C14	2.7 (2)	K1—N9—C59—C60	9.6 (2)
C11—N3—C14—C15	−173.10 (19)	C61—O5—C60—C59	−150.8 (3)
Fe1—N3—C14—C15	5.8 (3)	C61A—O5—C60—C59	−174.6 (4)
C11—N3—C14—C13	−0.2 (2)	K1—O5—C60—C59	80.20 (18)
Fe1—N3—C14—C13	178.76 (13)	N9—C59—C60—O5	−68.4 (3)
C12—C13—C14—N3	−1.7 (2)	C60—O5—C61—C62	−81.0 (4)
C12—C13—C14—C15	171.39 (19)	K1—O5—C61—C62	35.4 (4)
N3—C14—C15—C16	9.5 (3)	C63—O6—C62—C61	−171.9 (3)
C13—C14—C15—C16	−162.42 (19)	K1—O6—C62—C61	68.4 (4)
N3—C14—C15—C35	−174.72 (18)	O5—C61—C62—O6	−70.4 (5)
C13—C14—C15—C35	13.4 (3)	C60—O5—C61A—C62A	−167.9 (8)
C19—N4—C16—C15	172.86 (18)	K1—O5—C61A—C62A	−73.3 (8)
Fe1—N4—C16—C15	−7.8 (3)	C63A—O6A—C62A—C61A	156.7 (10)
C19—N4—C16—C17	−1.9 (2)	K1—O6A—C62A—C61A	−60.9 (11)
Fe1—N4—C16—C17	177.47 (13)	O5—C61A—C62A—O6A	87.3 (11)
C14—C15—C16—N4	−8.4 (3)	C62—O6—C63—C64	−176.6 (3)
C35—C15—C16—N4	176.02 (18)	K1—O6—C63—C64	−58.3 (3)
C14—C15—C16—C17	165.5 (2)	C52—N10—C64—C63	78.9 (3)
C35—C15—C16—C17	−10.1 (3)	C58—N10—C64—C63	−157.4 (2)
N4—C16—C17—C18	3.6 (2)	K1—N10—C64—C63	−48.8 (3)
C15—C16—C17—C18	−171.05 (19)	O6—C63—C64—N10	73.6 (3)
C16—C17—C18—C19	−3.6 (2)	C62A—O6A—C63A—C64A	174.3 (11)
C16—N4—C19—C20	172.73 (18)	K1—O6A—C63A—C64A	29.1 (9)
Fe1—N4—C19—C20	−6.6 (3)	C58A—N10—C64A—C63A	−73.9 (11)
C16—N4—C19—C18	−0.4 (2)	C52A—N10—C64A—C63A	162.5 (8)
Fe1—N4—C19—C18	−179.73 (13)	K1—N10—C64A—C63A	56.7 (10)
C17—C18—C19—N4	2.6 (2)	O6A—C63A—C64A—N10	−59.4 (12)
C17—C18—C19—C20	−170.34 (19)	C77—N8—C65—C66	−175.0 (2)
N4—C19—C20—C1	−10.1 (3)	C71—N8—C65—C66	62.2 (3)
C18—C19—C20—C1	161.94 (19)	K2—N8—C65—C66	−62.4 (2)
N4—C19—C20—C29	176.30 (18)	C67—O9—C66—C65	164.6 (2)
C18—C19—C20—C29	−11.7 (3)	K2—O9—C66—C65	−0.6 (3)
N1—C1—C20—C19	9.4 (3)	N8—C65—C66—O9	48.0 (3)
C2—C1—C20—C19	−160.71 (19)	C66—O9—C67—C68	−130.8 (2)
N1—C1—C20—C29	−176.70 (18)	K2—O9—C67—C68	35.5 (2)
C2—C1—C20—C29	13.2 (3)	C69—O10—C68—C67	171.66 (18)
C4—C5—C21—C22	−116.6 (2)	K2—O10—C68—C67	59.52 (19)
C6—C5—C21—C22	60.1 (3)	O9—C67—C68—O10	−65.4 (2)
C4—C5—C21—C26	62.8 (2)	C68—O10—C69—C70	168.95 (18)
C6—C5—C21—C26	−120.5 (2)	K2—O10—C69—C70	−76.75 (17)
C26—C21—C22—C23	1.1 (3)	C76—N7—C70—C69	−146.31 (19)
C5—C21—C22—C23	−179.5 (2)	C82—N7—C70—C69	91.1 (2)
C21—C22—C23—C24	−0.4 (4)	K2—N7—C70—C69	−24.0 (2)
C22—C23—C24—C25	−0.2 (4)	O10—C69—C70—N7	71.3 (2)
C22—C23—C24—Cl2	−179.44 (19)	C77—N8—C71—C72	82.7 (2)
C23—C24—C25—C26	0.2 (4)	C65—N8—C71—C72	−154.6 (2)
Cl2—C24—C25—C26	179.40 (17)	K2—N8—C71—C72	−40.4 (3)
C24—C25—C26—C21	0.5 (3)	C73—O7—C72—C71	172.0 (2)
C22—C21—C26—C25	−1.1 (3)	K2—O7—C72—C71	−54.9 (2)
C5—C21—C26—C25	179.46 (18)	N8—C71—C72—O7	64.2 (3)
C19—C20—C29—C31	71.0 (3)	C72—O7—C73—C74	178.76 (19)
C1—C20—C29—C31	−103.1 (2)	K2—O7—C73—C74	46.7 (2)
C19—C20—C29—C30	−115.5 (2)	C75—O11—C74—C73	−173.67 (19)
C1—C20—C29—C30	70.5 (2)	K2—O11—C74—C73	54.9 (2)
C31—C29—C30—C34	−0.2 (3)	O7—C73—C74—O11	−68.4 (2)
C20—C29—C30—C34	−174.0 (2)	C74—O11—C75—C76	177.21 (19)
C30—C29—C31—C32	−1.1 (3)	K2—O11—C75—C76	−51.4 (2)
C20—C29—C31—C32	172.5 (2)	C70—N7—C76—C75	74.8 (2)
C29—C31—C32—C33	1.2 (3)	C82—N7—C76—C75	−163.33 (19)
C31—C32—C33—C34	0.1 (3)	K2—N7—C76—C75	−45.2 (2)
C31—C32—C33—Cl4	−179.37 (17)	O11—C75—C76—N7	67.1 (3)
C32—C33—C34—C30	−1.3 (3)	C71—N8—C77—C78	−161.6 (2)
Cl4—C33—C34—C30	178.14 (18)	C65—N8—C77—C78	75.0 (2)
C29—C30—C34—C33	1.4 (4)	K2—N8—C77—C78	−32.9 (2)
C16—C15—C35—C36	−120.4 (2)	C79—O12—C78—C77	177.40 (18)
C14—C15—C35—C36	63.7 (3)	K2—O12—C78—C77	−65.58 (19)
C16—C15—C35—C40	64.8 (3)	N8—C77—C78—O12	71.3 (3)
C14—C15—C35—C40	−111.2 (2)	C78—O12—C79—C80	174.91 (18)
C40—C35—C36—C37	0.9 (3)	K2—O12—C79—C80	59.15 (19)
C15—C35—C36—C37	−174.2 (2)	C81—O8—C80—C79	−175.28 (18)
C35—C36—C37—C38	0.1 (4)	K2—O8—C80—C79	41.7 (2)
C36—C37—C38—C39	−1.0 (4)	O12—C79—C80—O8	−69.6 (2)
C36—C37—C38—Cl1	178.22 (19)	C80—O8—C81—C82	172.15 (17)
C37—C38—C39—C40	0.9 (4)	K2—O8—C81—C82	−47.1 (2)
Cl1—C38—C39—C40	−178.35 (18)	C76—N7—C82—C81	73.2 (2)
C38—C39—C40—C35	0.2 (4)	C70—N7—C82—C81	−163.40 (19)
C36—C35—C40—C39	−1.0 (3)	K2—N7—C82—C81	−47.8 (2)
C15—C35—C40—C39	173.8 (2)	O8—C81—C82—N7	65.1 (2)
C11—C10—C41—C46	122.3 (2)	C86—O13—C83—C84	−9.3 (5)
C9—C10—C41—C46	−59.6 (3)	O13—C83—C84—C85	−9.1 (5)
C11—C10—C41—C42	−57.7 (3)	C83—C84—C85—C86	23.3 (5)
C9—C10—C41—C42	120.5 (2)	C83—O13—C86—C85	24.6 (5)
C46—C41—C42—C43	−0.3 (3)	C84—C85—C86—O13	−29.7 (5)
C10—C41—C42—C43	179.66 (19)	C90—O14—C87—C88	−27.0 (9)
C41—C42—C43—C44	−0.3 (3)	O14—C87—C88—C89	33.6 (10)
C42—C43—C44—C45	0.3 (3)	C87—C88—C89—C90	−28.1 (10)
C42—C43—C44—Cl3	−179.56 (16)	C87—O14—C90—C89	7.4 (8)
C43—C44—C45—C46	0.3 (3)	C88—C89—C90—O14	14.4 (10)
Cl3—C44—C45—C46	−179.84 (16)	C90A—O14A—C87A—C88A	−13.2 (18)
C42—C41—C46—C45	0.9 (3)	O14A—C87A—C88A—C89A	−10.2 (19)
C10—C41—C46—C45	−179.04 (19)	C87A—C88A—C89A—C90A	29.3 (19)
C44—C45—C46—C41	−0.9 (3)	C87A—O14A—C90A—C89A	33.6 (19)
C53—N9—C47—C48	−169.56 (18)	C88A—C89A—C90A—O14A	−39.6 (19)
C59—N9—C47—C48	65.7 (2)		
